# Using the WHO Health Equity Assessment Toolkit to investigate changes in rural vs urban malnutrition inequality for low- and middle-income countries

**DOI:** 10.1186/s12939-026-02798-y

**Published:** 2026-04-21

**Authors:** Patrick C. Roney, Anca D. Dragomir, George Luta

**Affiliations:** 1 Department of Biostatistics, Bioinformatics, and Biomathematics, Georgetown University Medical Center, 4000 Reservoir Rd NW, Washington DC, 20057 USA; 2 Department of Oncology, Georgetown University Medical Center, 3900 Reservoir Rd NW, Washington DC, 20057 USA

**Keywords:** Childhood malnutrition, Childhood health, Low- and middle-income countries

## Abstract

**Background:**

Reducing health inequalities is a key element in the World Health Organization’s (WHO) sustainable development goals (SDG). While previous research has analyzed individual childhood malnutrition indicators and inequality by place of residence, there are no studies that have looked over time for rural vs. urban inequalities for all five-childhood malnutrition indicators for low- and middle-income countries (LMIC). The goal of the current study is to provide preliminary results to address this research gap by using a free software developed by WHO, the Health Equity Assessment Toolkit (HEAT).

**Methods:**

The 52 countries included in this study had at least two Demographics and Health Survey (DHS) datasets, between 1990 and 2021, available through the WHO HEAT. All five childhood malnutrition indicators (overweight, stunting, underweight, wasting, and severe wasting) were evaluated. An increase in inequality was considered to be an increase over time in the difference between the rural prevalence and the urban prevalence of a malnutrition indicator.

**Results:**

Out of the 52 countries, 15 (29%) reduced inequality across all five malnutrition indicators, 13 (25%) reduced inequality for 4 indicators, 7 (13%) reduced inequality for 3 indicators, 7 (13%) reduced inequality for 2 indicators, 7 (13%) reduced inequality for one indicator, and 3 (6%) did not reduce inequality for any indicator. The overweight indicator had the most countries (42%) showing an increase in inequality, while the underweight indicator had the fewest countries (19%) showing an increase in inequality.

**Conclusions:**

Overall, most LMIC were showing progress towards reducing malnutrition inequality based on place of residence. Results show that malnutrition inequality is generally decreasing for LMIC, with the exception of the overweight indicator. To further understand what is driving the observed inequalities and inform policy changes, future analyses should evaluate possible drivers such as economic transitions, urbanization rates, or health policy changes. WHO HEAT software provides free and easy access to relevant survey data and provides summary statistics and plots that allow the user to understand the data and answer a variety of inequality-related research questions.

**Supplementary Information:**

The online version contains supplementary material available at 10.1186/s12939-026-02798-y.

## Background

The sustainable development goals (SDG) from the World Health Organization (WHO) emphasize the need for all countries to have better health while “leaving no one behind [1, 2]. While this new initiative includes all countries in the action items, health outcomes in low- and middle-income countries (LMIC) still require more research to provide evidence-based guidance on how the available resources should be used efficiently to improve outcomes and reduce inequality, or the “observed differences between subgroups of a population.” [3] One health outcome that needs to be understood more is childhood malnutrition, and an important research topic concerns malnutrition inequalities due to place of residence for children under the age of five. To our knowledge, there are no research studies that have considered all five malnutrition indicators. The goal of the current study is to provide preliminary results to address this research gap by using a free software developed by WHO, which makes exploring global health topics easy.

Previous research work on national childhood malnutrition typically has focused on only one malnutrition indicator when considering all LMIC. For stunting, one of the five malnutrition indicators, several previous studies found that children from rural areas had higher odds of stunting compared to children from urban areas [4–8]. By contrast, the prevalence of being overweight was found either not to have an association with place of residence [9] or to be higher in urban areas [10]. Children from rural areas also have been found to have higher odds of severe wasting [10].

Previous research work has also noted the practical difficulties of finding centralized databases that may be used to calculate and report on inequalities on a subnational scale, and have also specified the need of having such data available for the construction of graphs and other forms of visual representation [11]. The Health Equity Assessment Toolkit (HEAT), an R shiny web application developed by the WHO, directly addresses this need since it provides access to a wealth of data repositories for a large number of countries, easily generates data summary visualizations, and also allows the possibility of downloading data for additional analysis using another software of the user’s choosing [3]. The data available in HEAT can be analyzed either in aggregate or by six different dimensions of inequality: age, economic status, education, place of residence, subnational region, and sex. It is important to note that for the current study the focus is on the place of residence, but the other dimensions of inequality are equally important even though they are not included in the current work. Since the release of HEAT in 2016, many researchers from around the world have used the free software to investigate a variety of research topics related to health inequalities, including research on child malnutrition inequality in the Uttar Pradesh state of India [12], on severe wasting inequality in Ethiopia [13], and on COVID-19 inequalities [14]. Studies like these have investigated the inequalities in childhood malnutrition related to place of residence for an individual country. To our knowledge, our study is the first research study that uses HEAT to aggregate data from all LMICs with the goal of evaluating inequality trends for all five childhood malnutrition indicators. Our work is intended to be a preliminary step in advancing current knowledge and guiding future research directions by generating new questions and hypotheses.

## Methods

Summary statistics from the Demographics Health Surveys (DHS), which are nationally-representative household surveys that provide data for a wide range of monitoring and impact evaluation indicators in the areas of population health and nutrition, were downloaded from HEAT. More details about the DHS methodology may be found on their website [15]. We studied five malnutrition indicators for children less than 5 years old, specifically the prevalence of the following: being overweight (i.e. being more than two standard deviations above the median weight-for-height of the WHO Child Growth Standards), severe wasting (being more than three standard deviations below the median weight-for-height of the WHO Child Growth Standards), stunting (being more than two standard deviations below the median height-for-age of the WHO Child Growth Standards), being underweight (being more than two standard deviations below the median weight-for height of the WHO Child Growth Standards), and wasting (being more than two standard deviations below the median weight-for-height of the WHO Child Growth Standards). The reader is referred to the HEAT User Manual [16] and HEAT technical notes [17] for details about the software capabilities and the related statistical methodology.

**Fig. 1:**
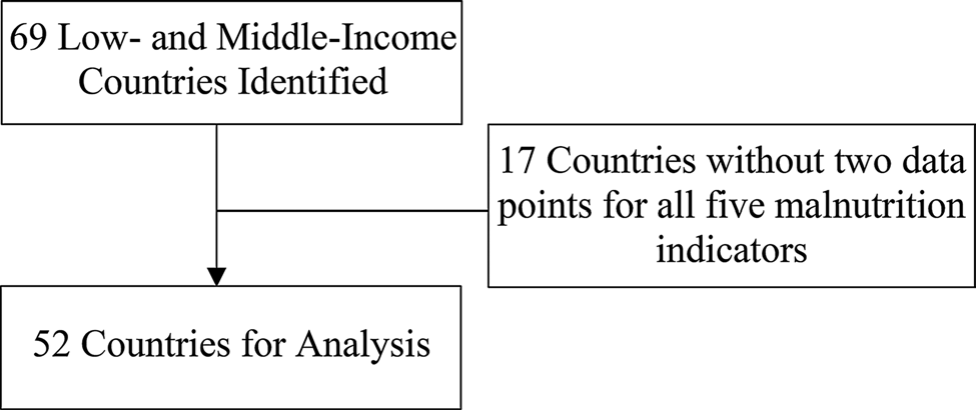
CONSORT diagram

After downloading from HEAT, the DHS data from 1990 to 2021 for 69 LMIC countries were further selected using R [18] so that the final analysis and plots created using ggplot2 [19] included the 52 LMICs with at least two DHS data timepoints available for all five malnutrition indicators (Fig. 1). Differences in prevalences of these malnutrition indicators between rural and urban areas were calculated for the earliest and most recent survey data. Differences between the prevalence difference at the most recent time point and the prevalence difference at the earliest time point were calculated to evaluate changes across time, together with corresponding 95% confidence intervals constructed using the standard errors provided by HEAT for the prevalence differences at the two time points. To compare countries across varying time intervals between earliest and most recent time points (4–29 years), we calculated the rate of change per year, by dividing the change by the number of years between the two time points. The construction of 95% confidence intervals is based on the formulas below:

$$\eqalign{ s.e.& \left({{{\hat \mu }_{recent}} - {{\hat \mu }_{earliest}}} \right) \cr & = \sqrt {s.e.{{\left({{{\hat \mu }_{recent}}} \right)}^2} + s.e.{{\left({{{\hat \mu }_{earliest}}} \right)}^2}} \cr}$$

$$\eqalign{ 95\% CI & = \left({{{\hat \mu }_{recent}} - {{\hat \mu }_{earliest}}} \right) \cr & \pm 1.96*s.e.\left({{{\hat \mu }_{recent}} - {{\hat \mu }_{earliest}}} \right) \cr}$$

**Fig. 2:**
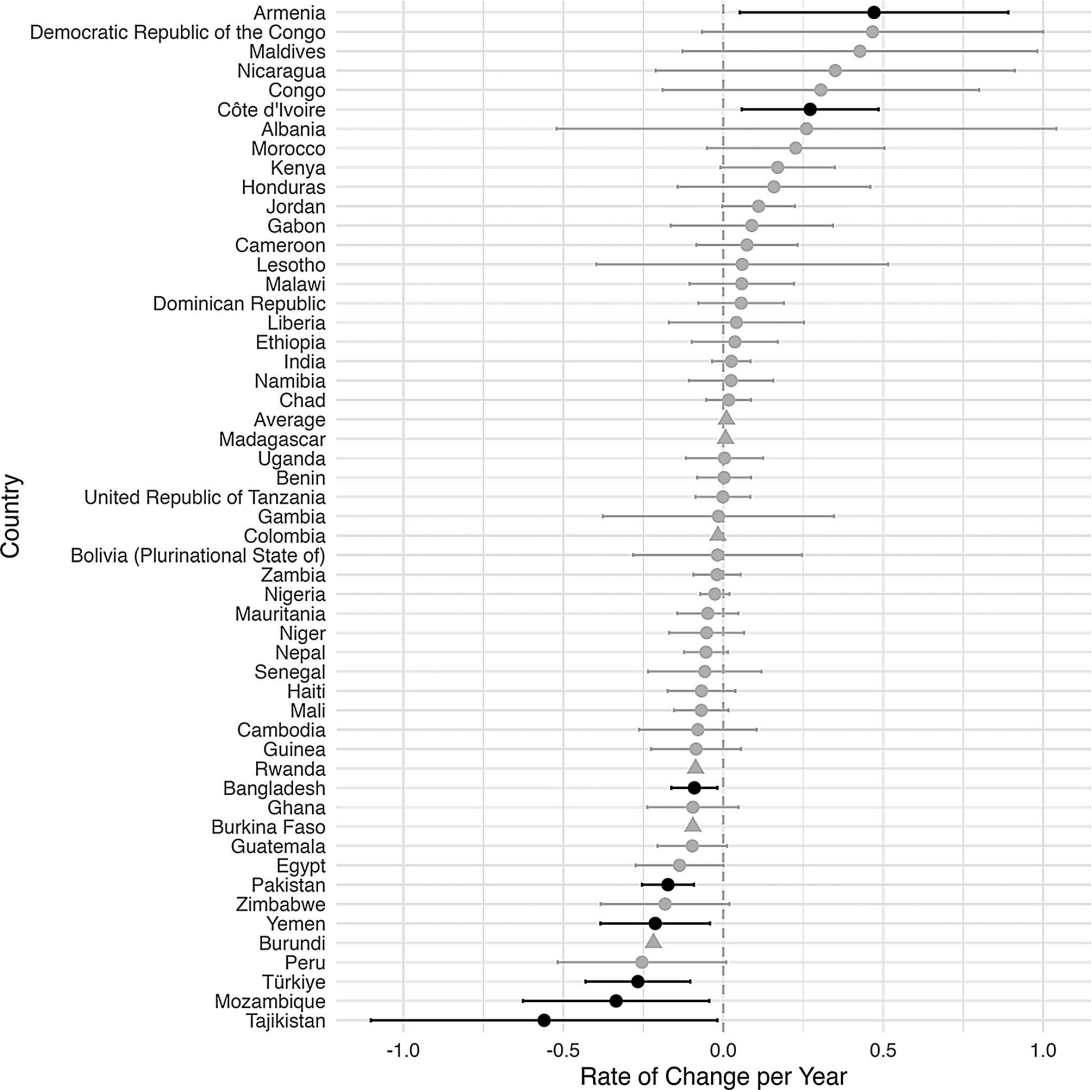
Change in rural-urban prevalence difference from earliest to recent time point for overweight indicator

**Fig. 3:**
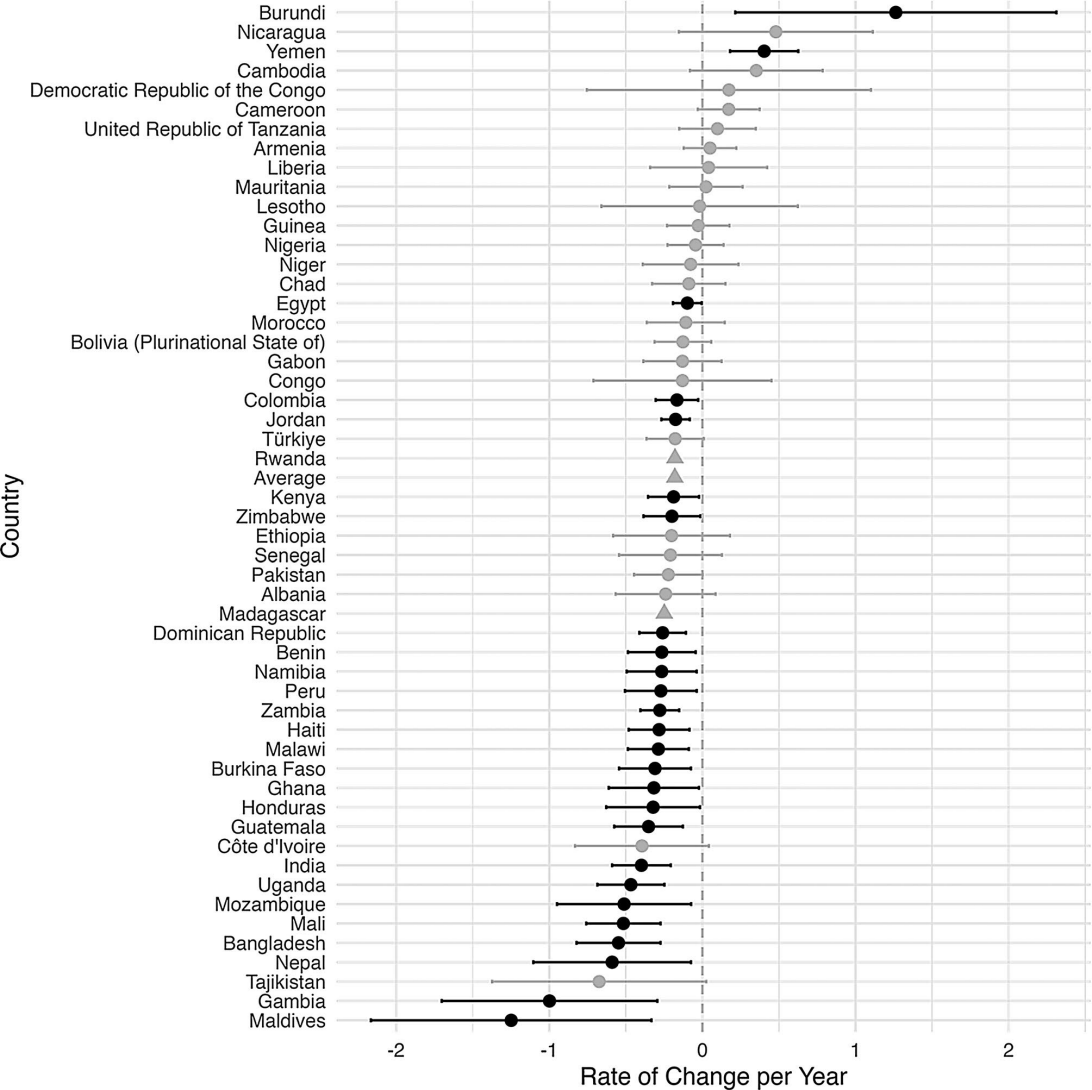
Change in rural-urban prevalence difference from earliest to recent time point for underweight indicator

**Fig. 4:**
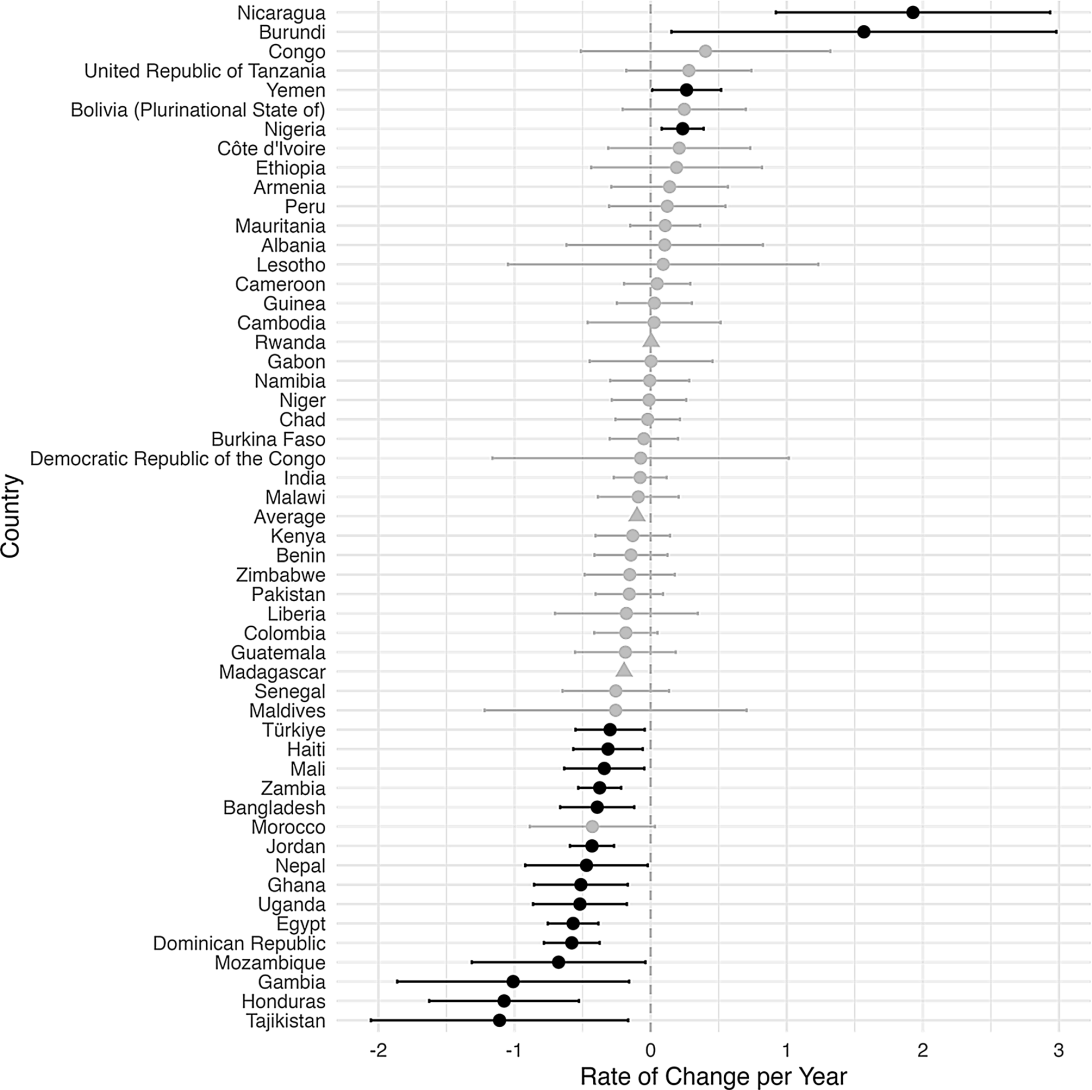
Change in rural-urban prevalence difference from earliest to recent time point for stunting indicator

**Fig. 5:**
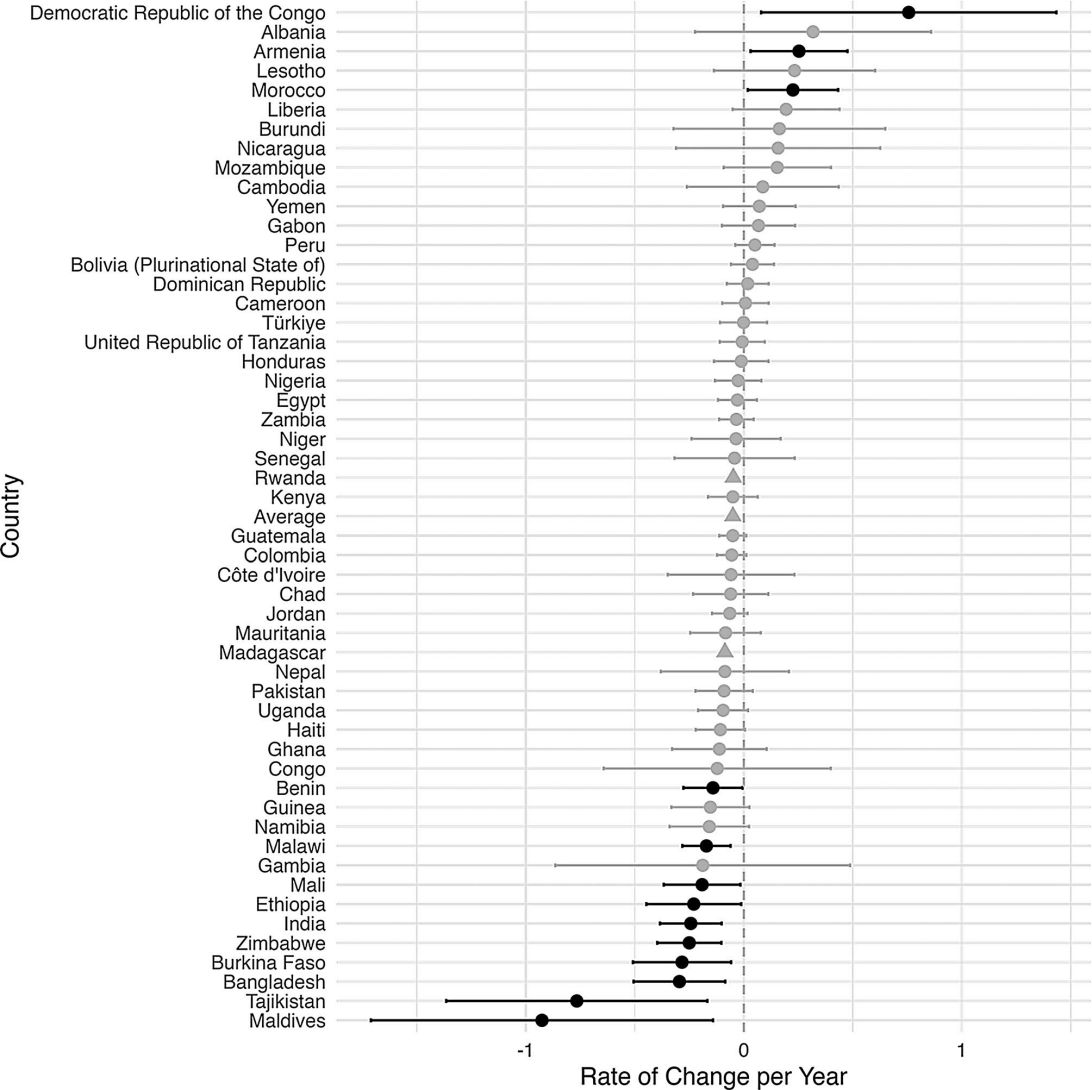
Change in rural-urban prevalence difference from earliest to recent time point for wasting indicator

**Fig. 6:**
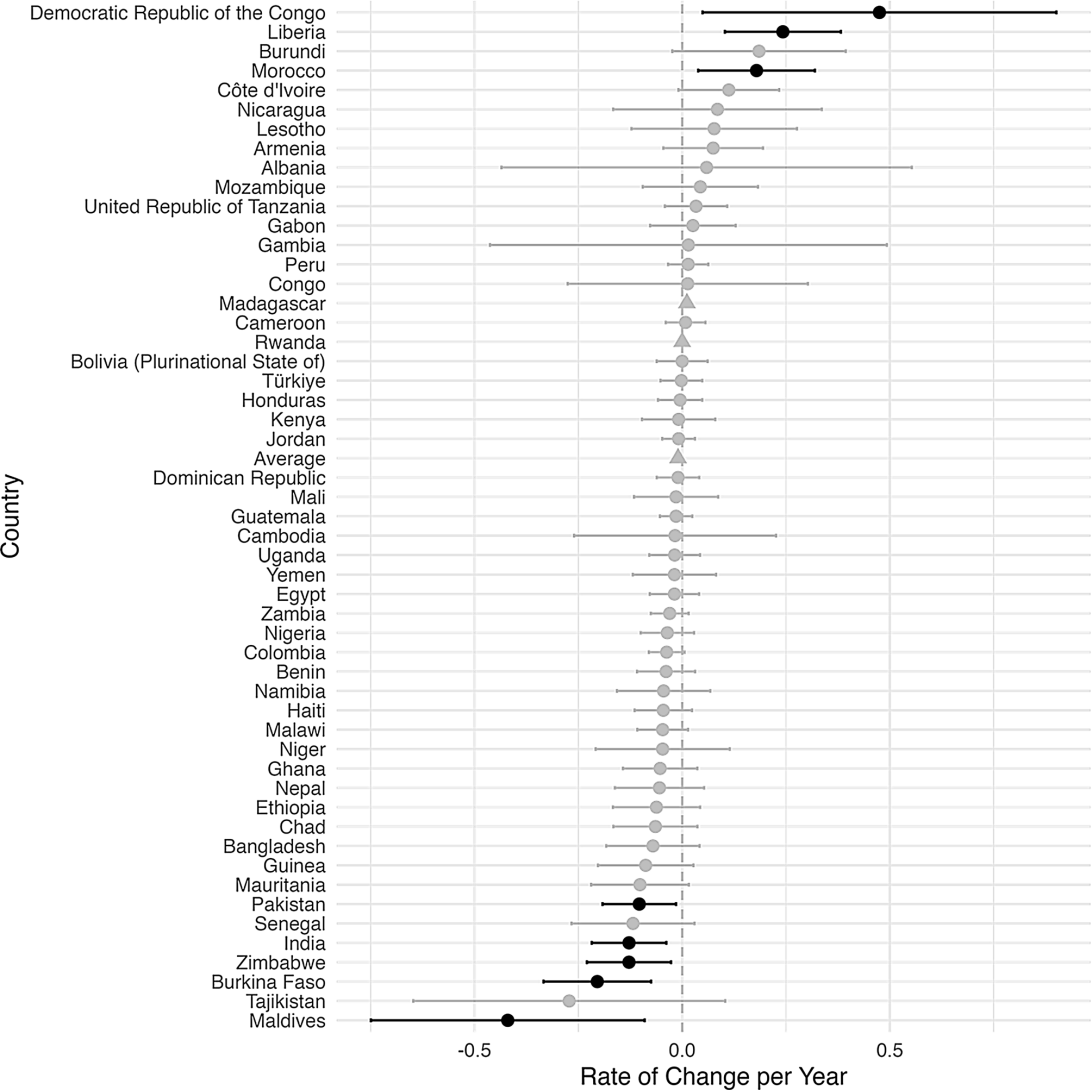
Change in rural-urban prevalence difference from earliest to recent time point for severe wasting indicator

An increase in inequality was defined to be an increase over time in the difference between the rural prevalence and the urban prevalence of a malnutrition indicator. Figures 2, 3, 4, 5 and 6, created using ggplot2, indicate the estimated inequality differences and 95% CIs, with gray lines indicating the situations when zero (the value indicating no true change in inequality) is included in the 95% confidence interval. Countries that did not have a 95% confidence interval calculated due to HEAT not providing a standard error are depicted with a triangle symbol, and the average was calculated as the mean across all countries in the figure.

Table 1 reports the estimated inequality difference over time and 95% CI. Dark red cells show a significant (95% CI not including 0) increase in inequality, light red cells show a non-significant increase in inequality, grey cells show negligible change (absolute value of the difference between time points less than 0.0049% points), light blue cells show a non-significant decrease in inequality, and dark blue cells show a significant increase in inequality. The rural-urban malnutrition inequalities with estimated inequality and 95% CI are reported for earliest time point (Table 2) and most recent time point (Table 3). Dark red cells show a significantly greater prevalence of the malnutrition indicator in rural areas (95% CI not including 0), light red cells show non-significant greater prevalence in rural areas, grey cells show negligible difference in prevalence (absolute value of the difference less than 0.0049% points), light blue cells show non-significant prevalence in urban areas, and dark blue cells show significantly greater prevalence in urban areas.

**Table 1:**
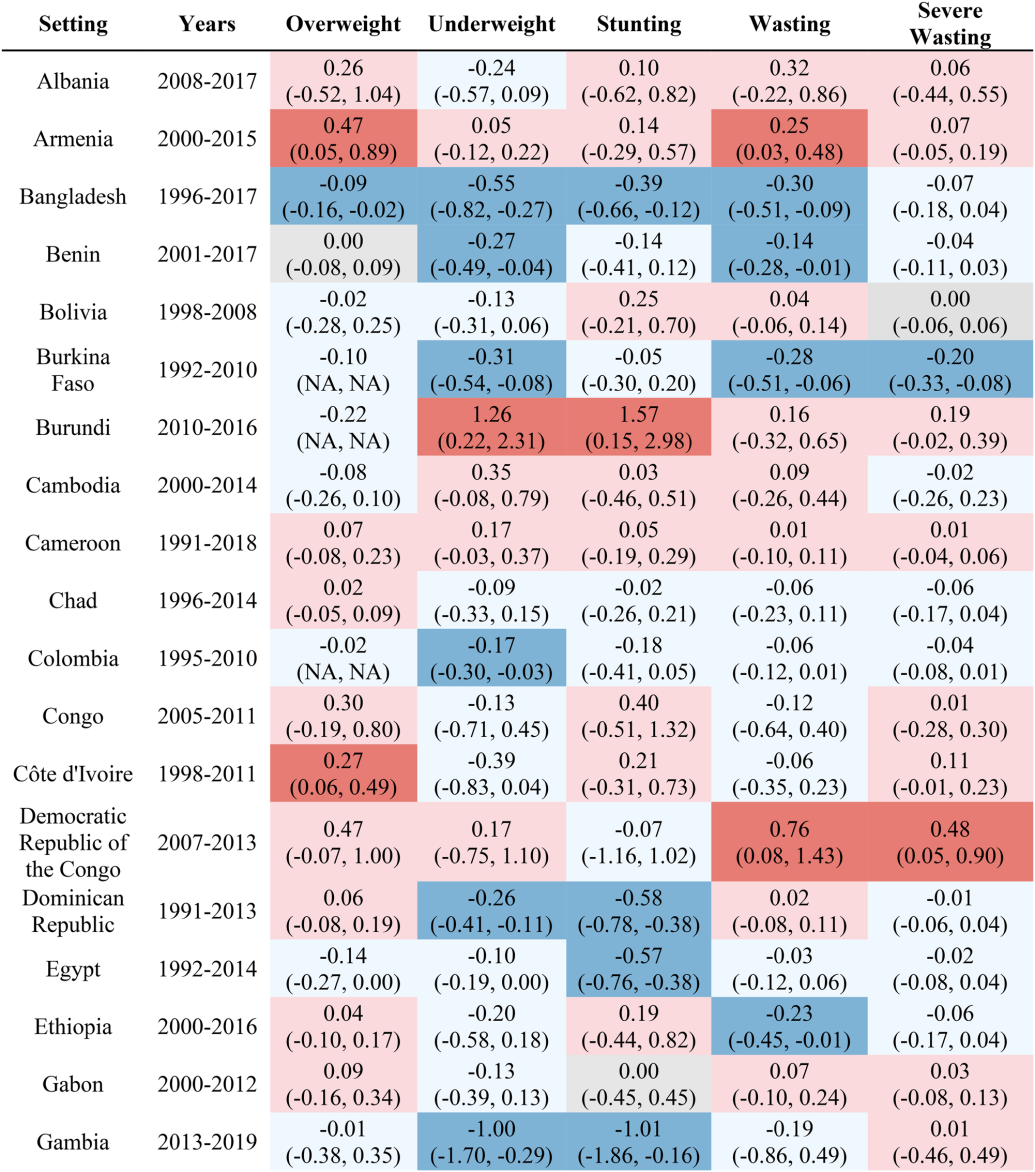

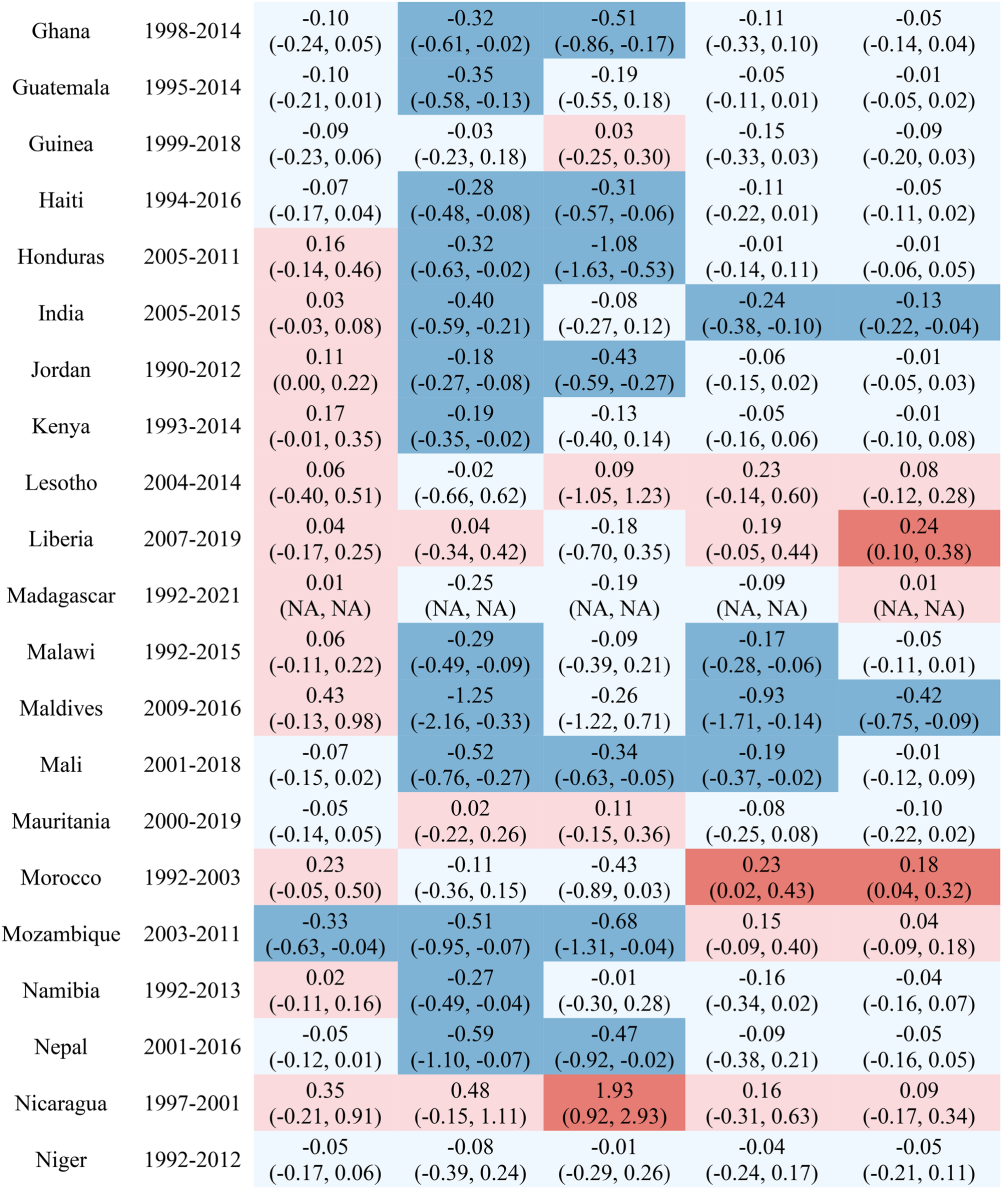

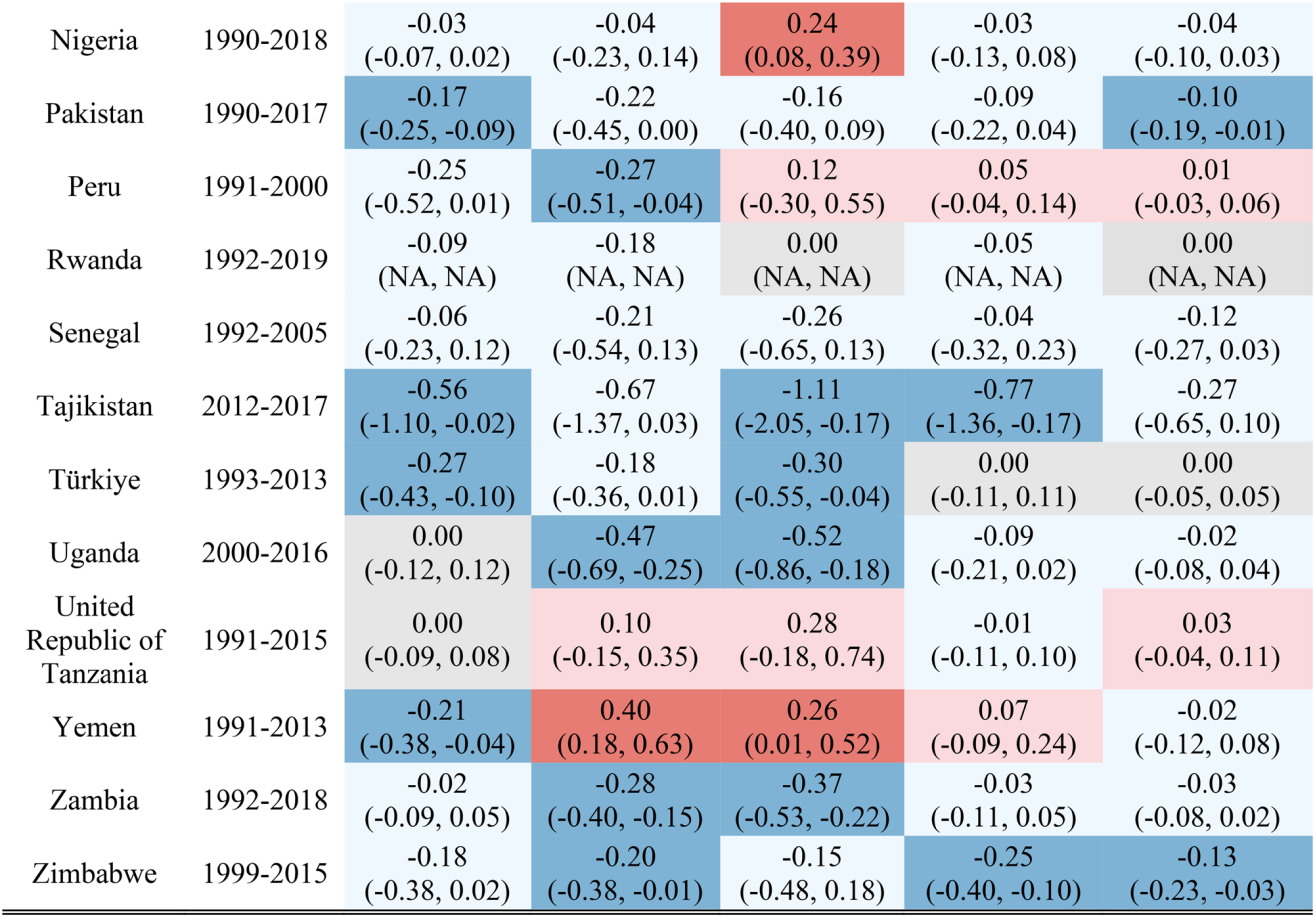
Countries, range of available years of DHS data, and indicator trends over time

**Table 2:**
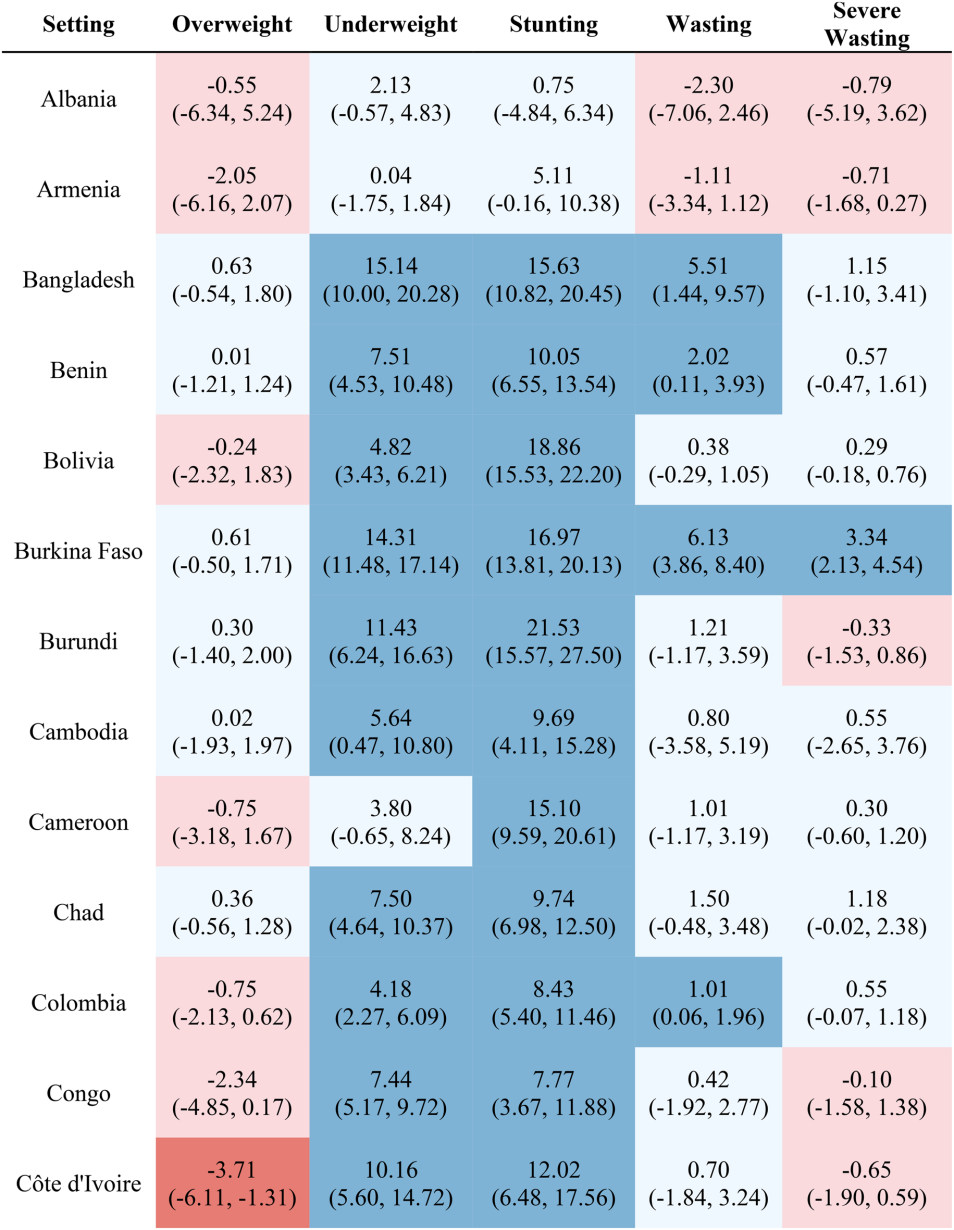

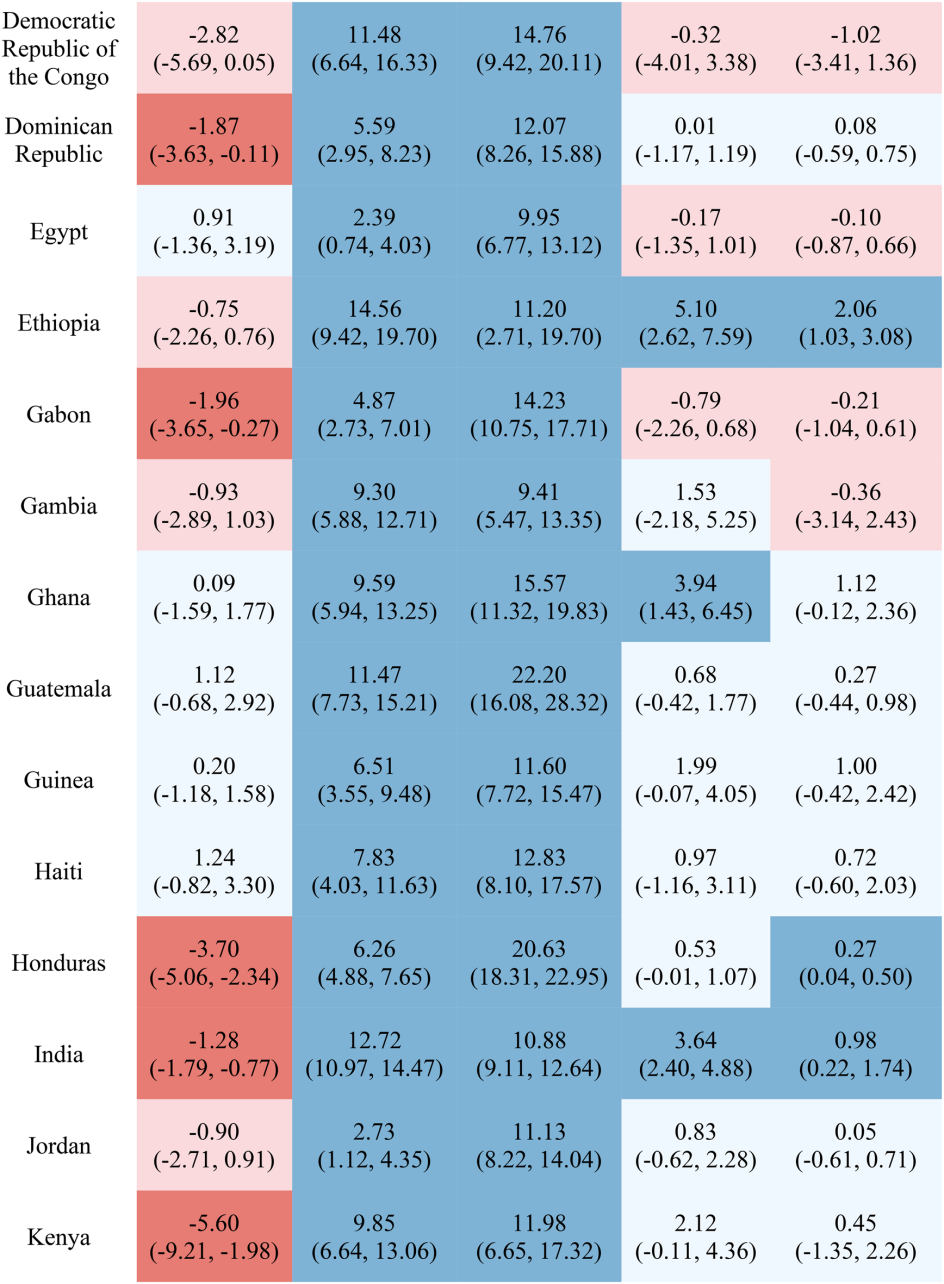

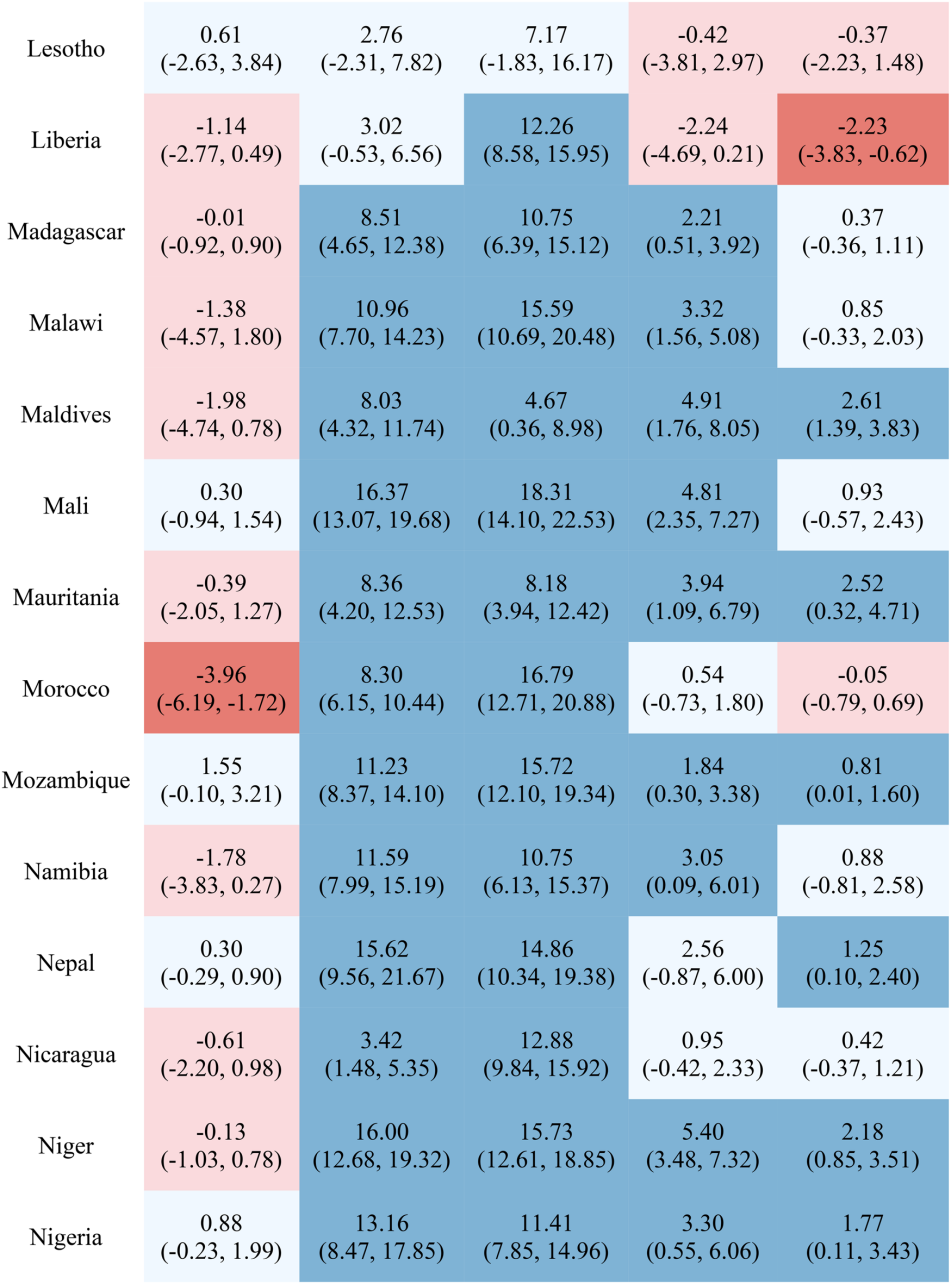

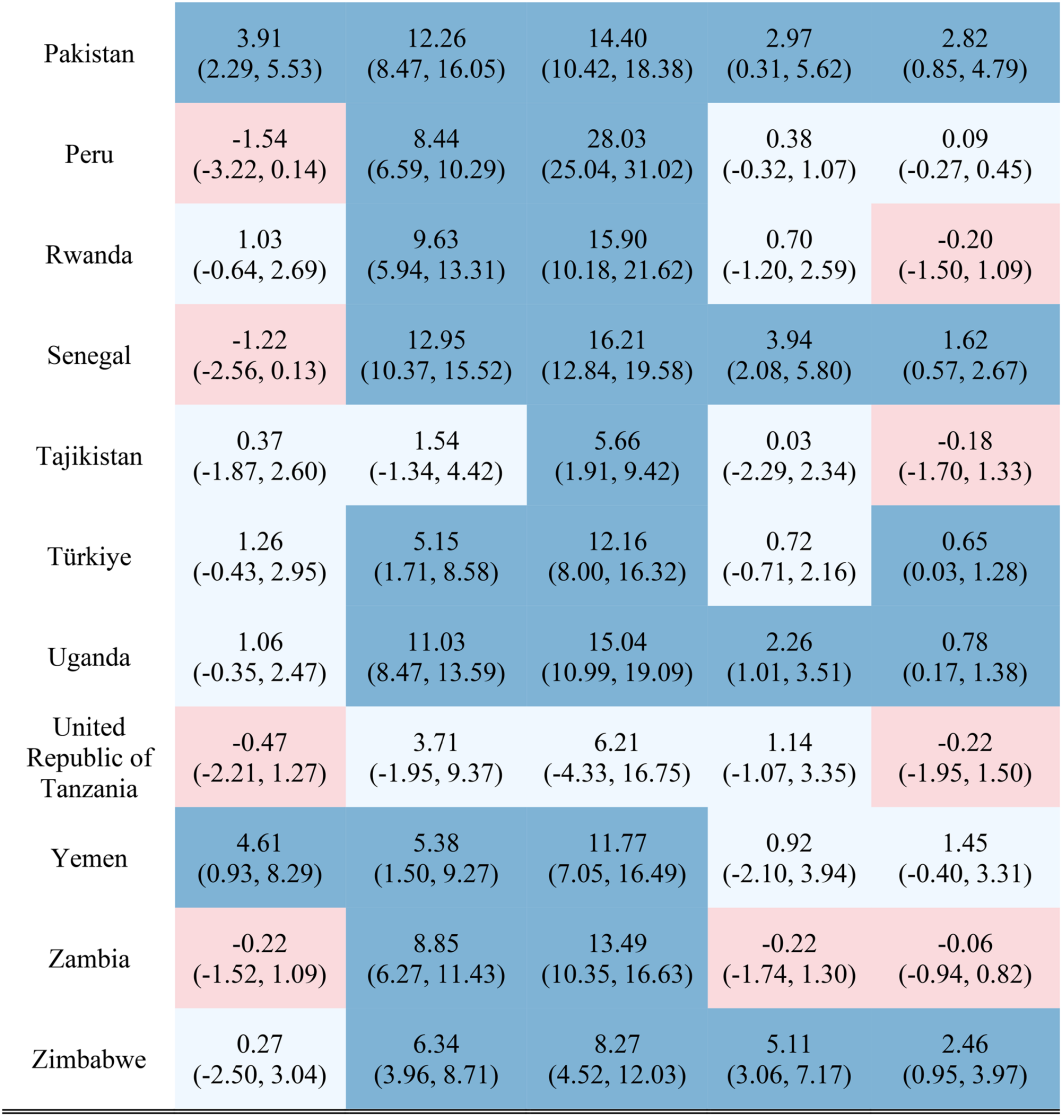
Countries and direction of inequality for earliest data point

**Table 3:**
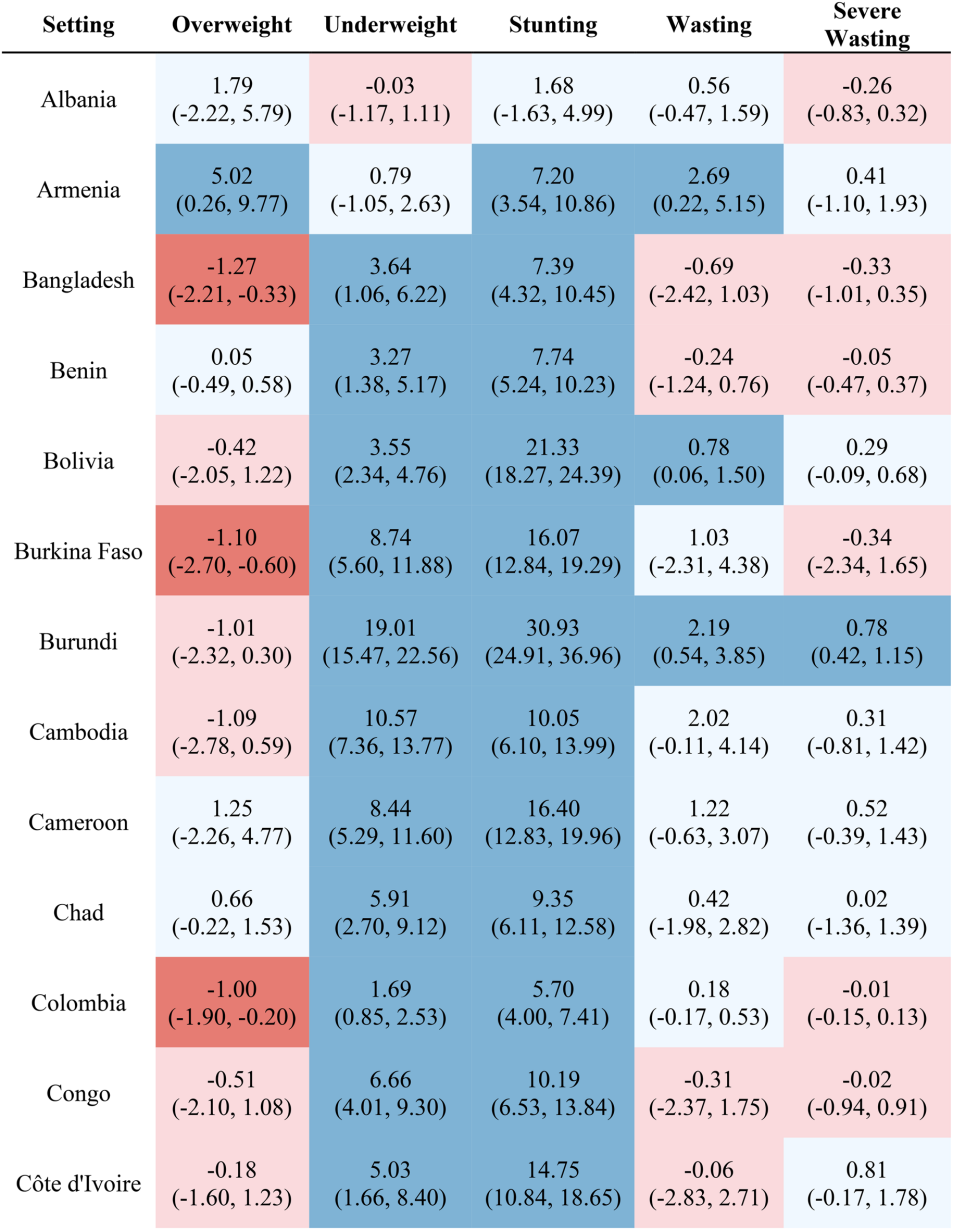

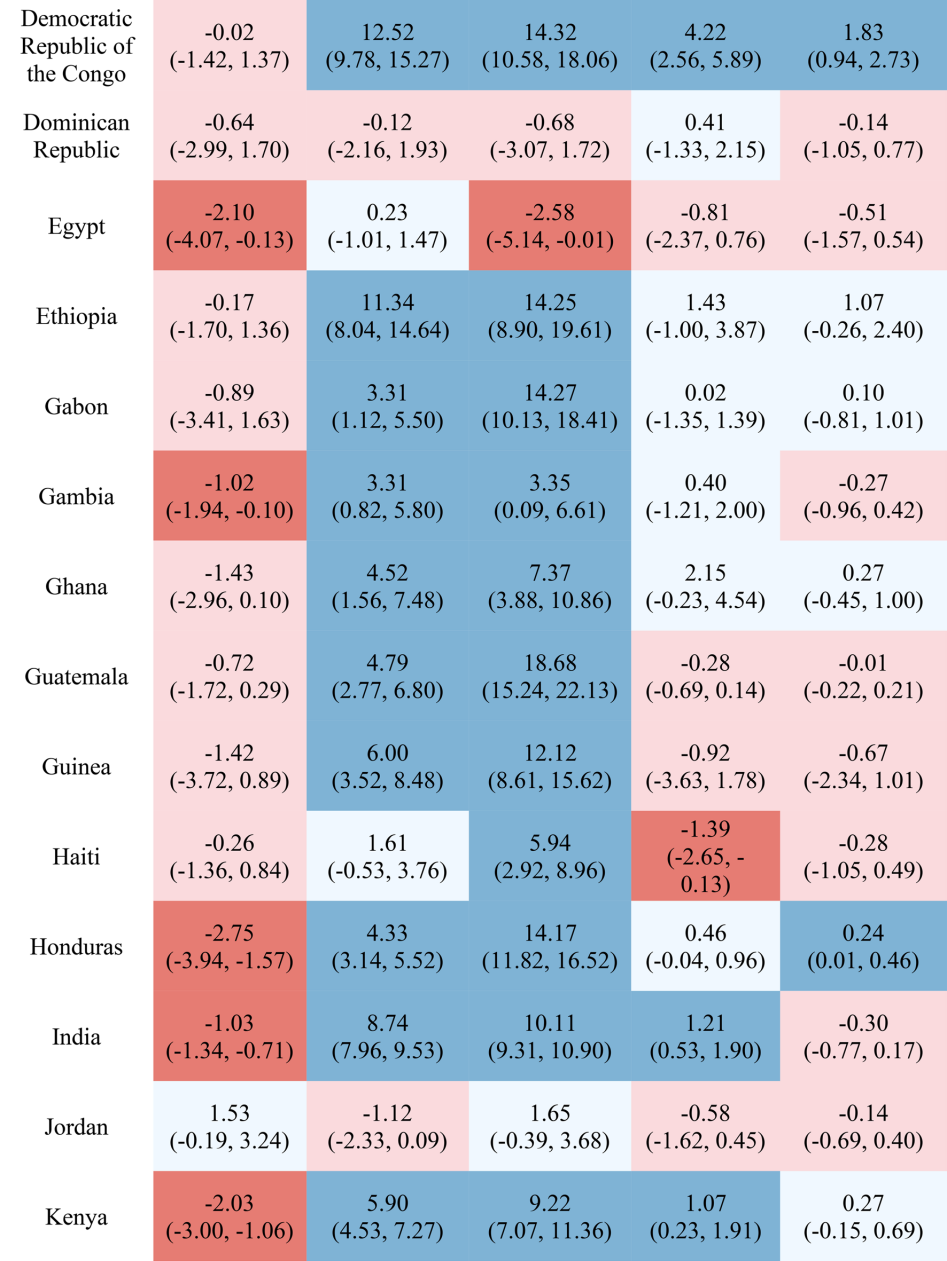

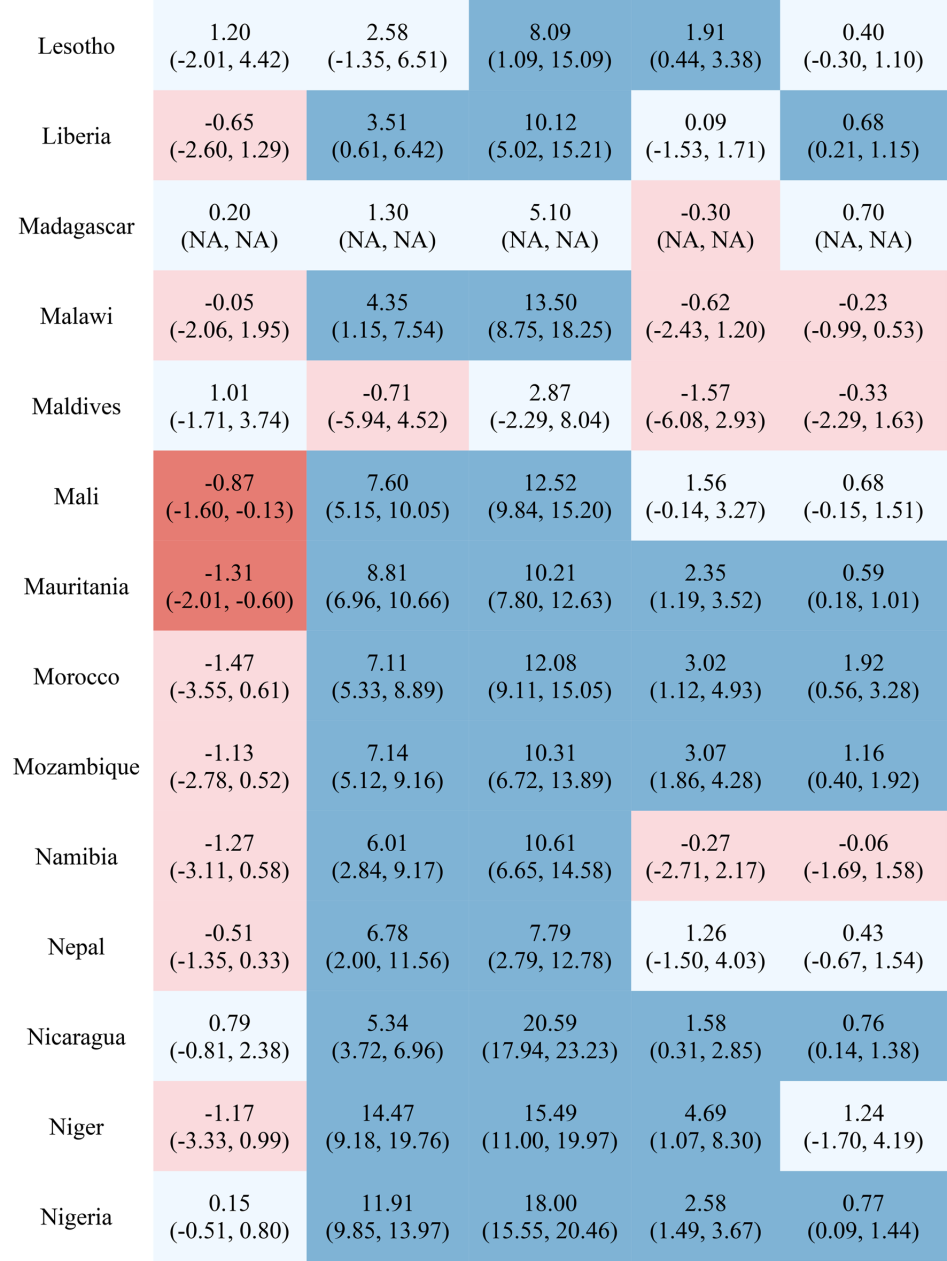

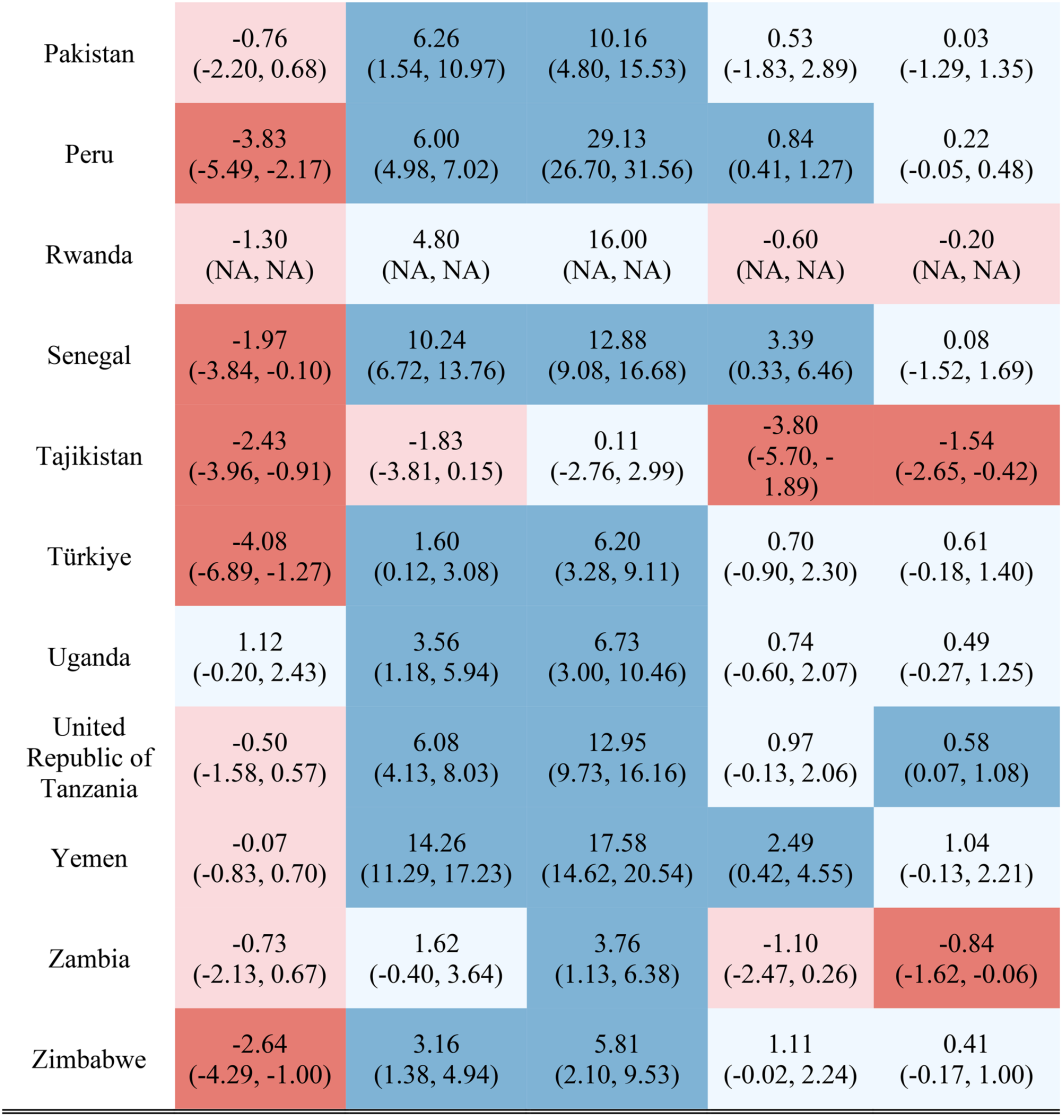
Countries and direction of inequality for most recent data point

Adjustment for potential confounding was not considered since we only report exploratory descriptive results. Future research should involve adjustment for country-level confounders, such as nutrition-related policies, as recommended by subject matter experts.

## Results

Out of the 52 countries, 15 (29%) reduced inequality across all five malnutrition indicators, 13 (25%) reduced inequality for 4 indicators, 7 (13%) reduced inequality for 3 indicators, 7 (13%) reduced inequality for 2 indicators, 7 (13%) reduced inequality for one indicator, and 3 (6%) did not reduce inequality for any indicator. Most countries (42%) showed an increase in inequality for the overweight indicator, while the underweight indicator showed an increase in inequality for the fewest countries (19%). It is notable that 28 of the 52 countries reduced inequality in at least four out of the five malnutrition indicators, while 8 countries, specifically Albania, Armenia, Burundi, Cameroon, the Democratic Republic of the Congo, Lesotho, Liberia, and Nicaragua, had an increase in inequality for at least four out of the five malnutrition indicators. (Table 1). Countries and direction of rural-urban inequality for earliest data point (Table 2) and most recent data point (Table 3) are also provided.

Figures S1 through S10 show the estimated prevalence difference between rural and urban areas at the earliest and most recent survey measurements with corresponding 95% confidence intervals. Stunting had the greatest inequality magnitude, as nearly all countries had a higher prevalence in rural areas compared to urban areas, with some countries having as much as a 30% points estimated difference in stunting prevalence. Similarly, for the underweight indicator, the majority of countries had a noticeably greater prevalence in rural areas compared to urban areas, although the difference was at most 20% points. The overweight indicator was the only malnutrition indicator to have greater prevalence in the urban areas than in the rural areas, although this indicator and the wasting and severe wasting indicators in general had similar prevalence values based on the place of residence. In addition, severe wasting in general had estimated prevalence differences around zero, while wasting tended to have greater prevalence in rural areas compared to urban areas.

Figures 2, 3, 4, 5 and 6 show the inequality difference with 95% confidence intervals between the most recent and the earliest surveys. It is important to note that Tajikistan consistently showed the most improvement across all the malnutrition indicators, since for every malnutrition indicator it was in the top three for the most reduction in inequality. The underweight indicator had the greatest average (mean) reduction at 1.85% points per year, while the overweight indicator was the only malnutrition indicator to have a slight increase of 0.05% points per year.

## Discussion

Compared with other significant work [20, 21] in this general research area, our study is complementary in the following way: it uses a free software to analyze publicly available data, and constitutes the first step of research on trends in malnutrition indicators across time. The results from our study are consistent with the results from the literature, for example with the results from Fotso 2006 and Menon, et al. 2000 which show that countries typically have greater prevalence of childhood malnutrition in rural areas compared to urban areas. However, our analysis also provides additional information as to which LMICs have reduced inequality over time, when previous work, such as Fagbamigbe, et al. 2020 and Black, et al. 2013, only analyze one malnutrition indicator at a time. Over half of the countries included in our analysis had reduced inequality in at least four of the five malnutrition indicators. Our work also has identified eight specific countries which had increased inequality over time, and therefore may need extra resources and support in order to reverse these trends. While much progress has been made in reducing undernutrition-related indicators, the overweight malnutrition indicator has seen less improvement, combined with an increase in prevalence in urban areas. To further understand what is driving persistent inequalities in these countries and inform policy changes, future analyses should evaluate possible drivers such as economic transitions, urbanization rates, or health policy changes. In particular, since our work provides an important exploratory finding that the overweight inequality is increasing, further research work is needed by experts in the field to identify its causes.

The development of HEAT has significantly improved the availability of relevant data by providing a centrally located data source easily accessible to quickly generate and view summary statistics, and download data for subsequent analyses using specialized statistical software. In addition, the vast amount of data available allows for the evaluation of national level and global level trends to be estimated. Future research studies should take full advantage of HEAT by expanding the types of research questions that can be answered based on the variety of data available through HEAT. One limitation of the current analysis is that we have not used statistical models to estimate prevalence differences while adjusting for potential socio-economic or demographic confounders. This is clearly possible and it will be the subject of future research work. We have not performed those more complex statistical analyses, because the purpose of our investigation was to estimate and report trends over time based on the available survey data. An additional limitation is the presence of recall bias and measurement error in the collection of the DHS data.

## Conclusions

Overall, most LMICs have showed progress towards reducing malnutrition inequality related to place of residence, with the most improvement occurring for the underweight indicator and the least improvement occurring for the overweight indicator. Policies should focus on reducing overweight inequality, while continuing to reduce the inequality for the other four malnutrition indicators. Policymakers could make decisions after follow-up studies are performed, which can more effectively target interventions, such as those targeting both undernutrition and overweight in rapidly urbanizing LMIC contexts, or prioritizing resource allocation to the eight countries with worsening trends. For health equity researchers, the HEAT software provides free and seamless access to relevant survey datasets, and relevant summary statistics and plots that allow the user to better understand the data and answer a variety of research questions. The possibilities of using HEAT to answer relevant health equity questions are only limited by our imagination!

## Supplementary Information

Below is the link to the electronic supplementary material.

Supplementary Material 1

## Data Availability

All data is available through the Health Equity Assessment Toolkit (HEAT) developed by the World Health Organization (WHO).

## Abbreviations

WHO: World Health Organization
HEAT: Health Equity Assessment Toolkit
SDG: Sustainable Development Goals
LMIC: Low- and middle-income countries
DHS: Demographics Health Survey
