# Supplementary material for: Using the WHO Health Equity Assessment Toolkit to investigate changes in rural vs urban malnutrition inequality for low- and middle-income countries
Source: Int J Equity Health. 2026 Apr 21;25:107. doi: 10.1186/s12939-026-02798-y (PMC13097934; doi:10.1186/s12939-026-02798-y)

**Supplementary Materials**

Figure S1. Overweight rural-urban prevalence difference at earliest data point.


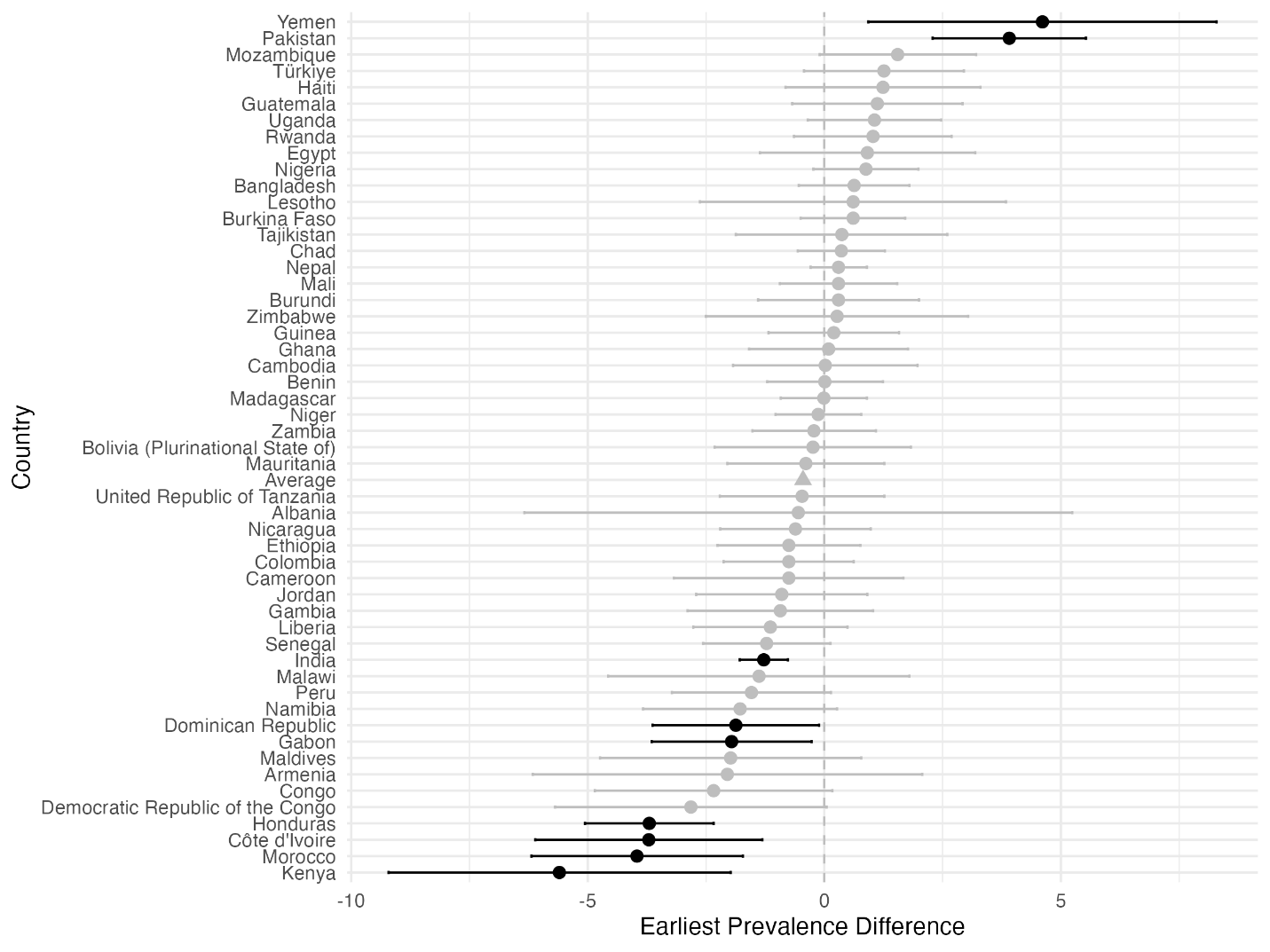


Figure S2. Overweight rural-urban prevalence difference at most recent data point.


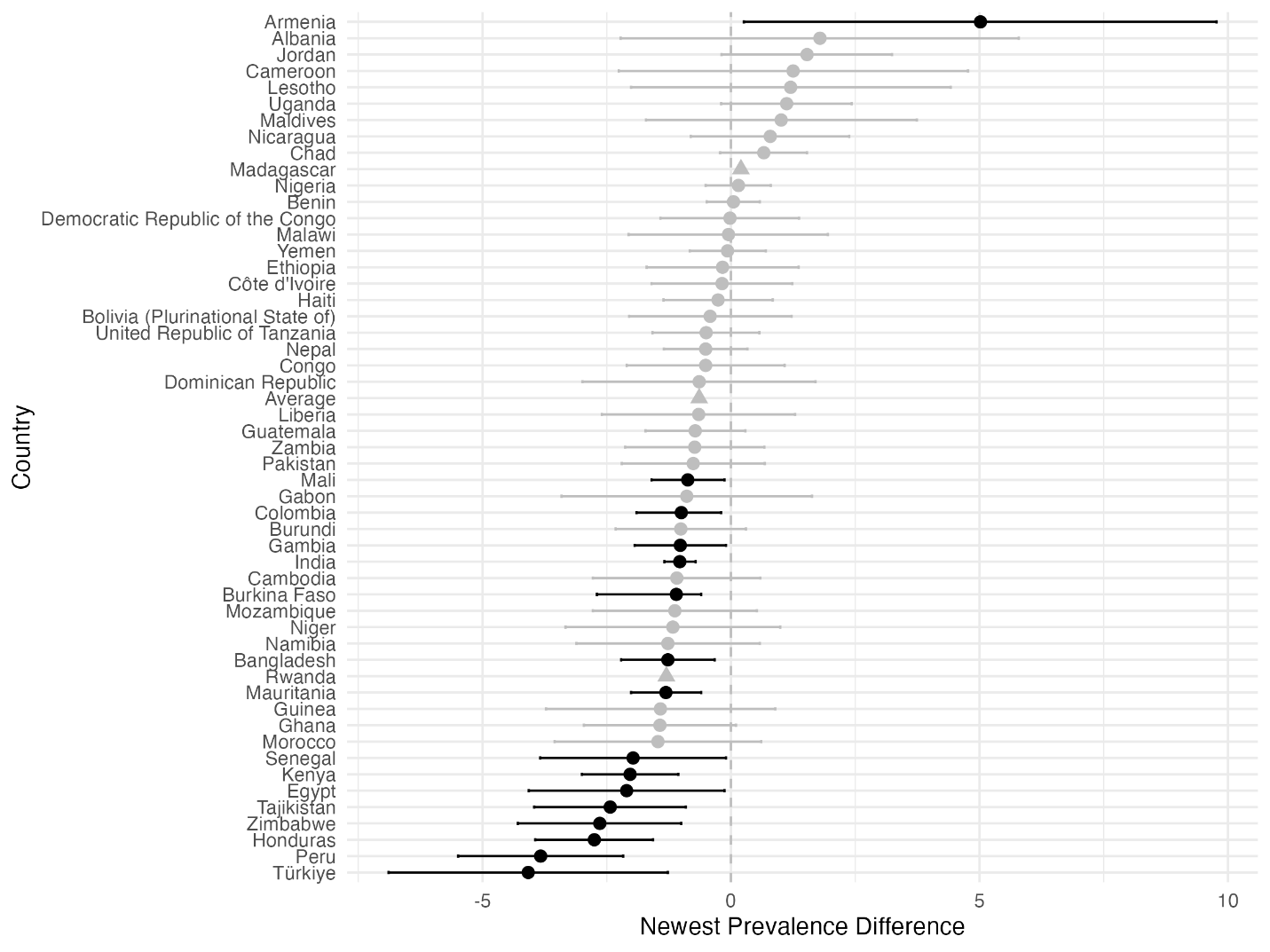


Figure S3. Underweight rural-urban prevalence difference at earliest data point.


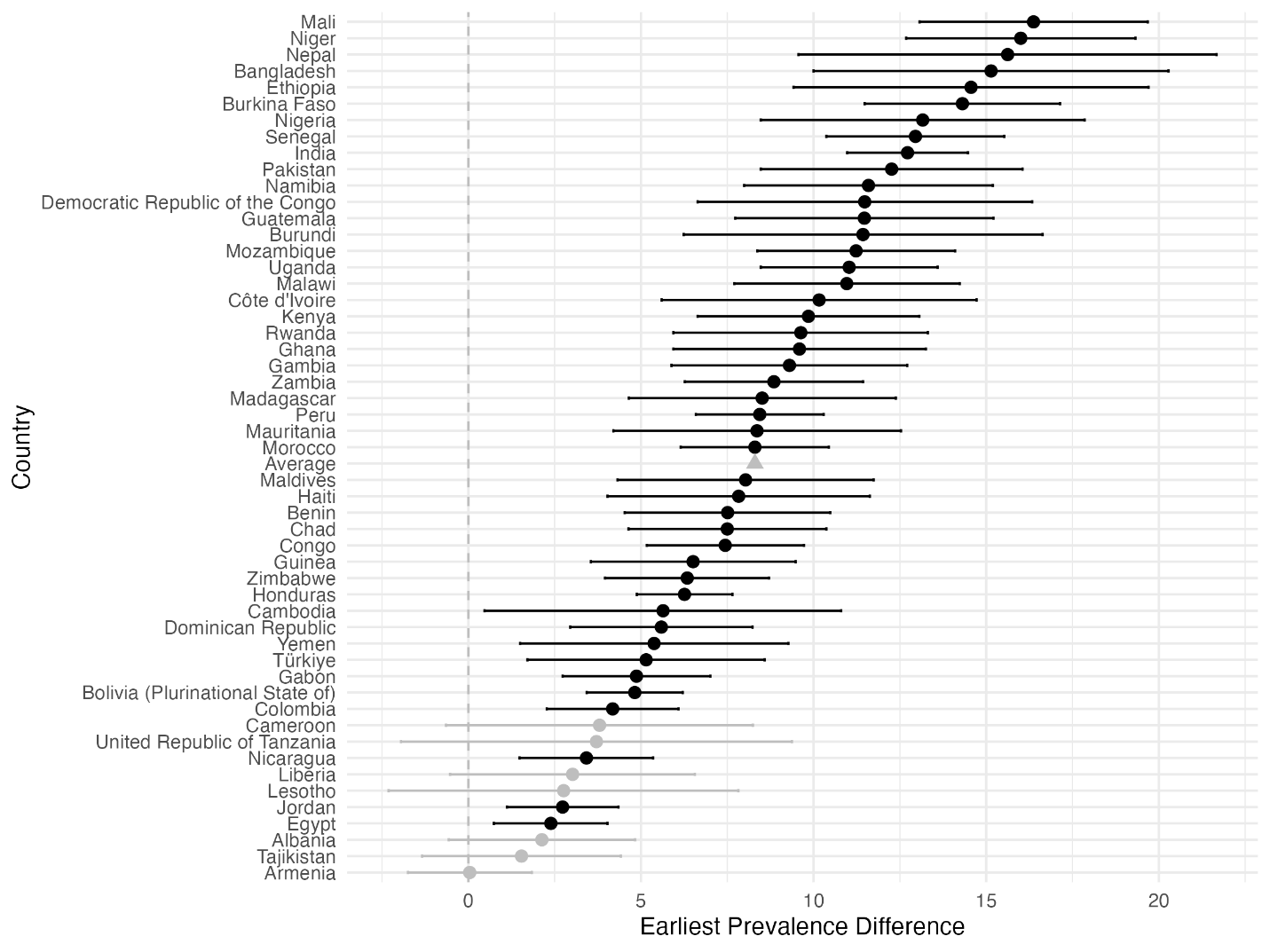


Figure S4. Underweight rural-urban prevalence difference at most recent data point.


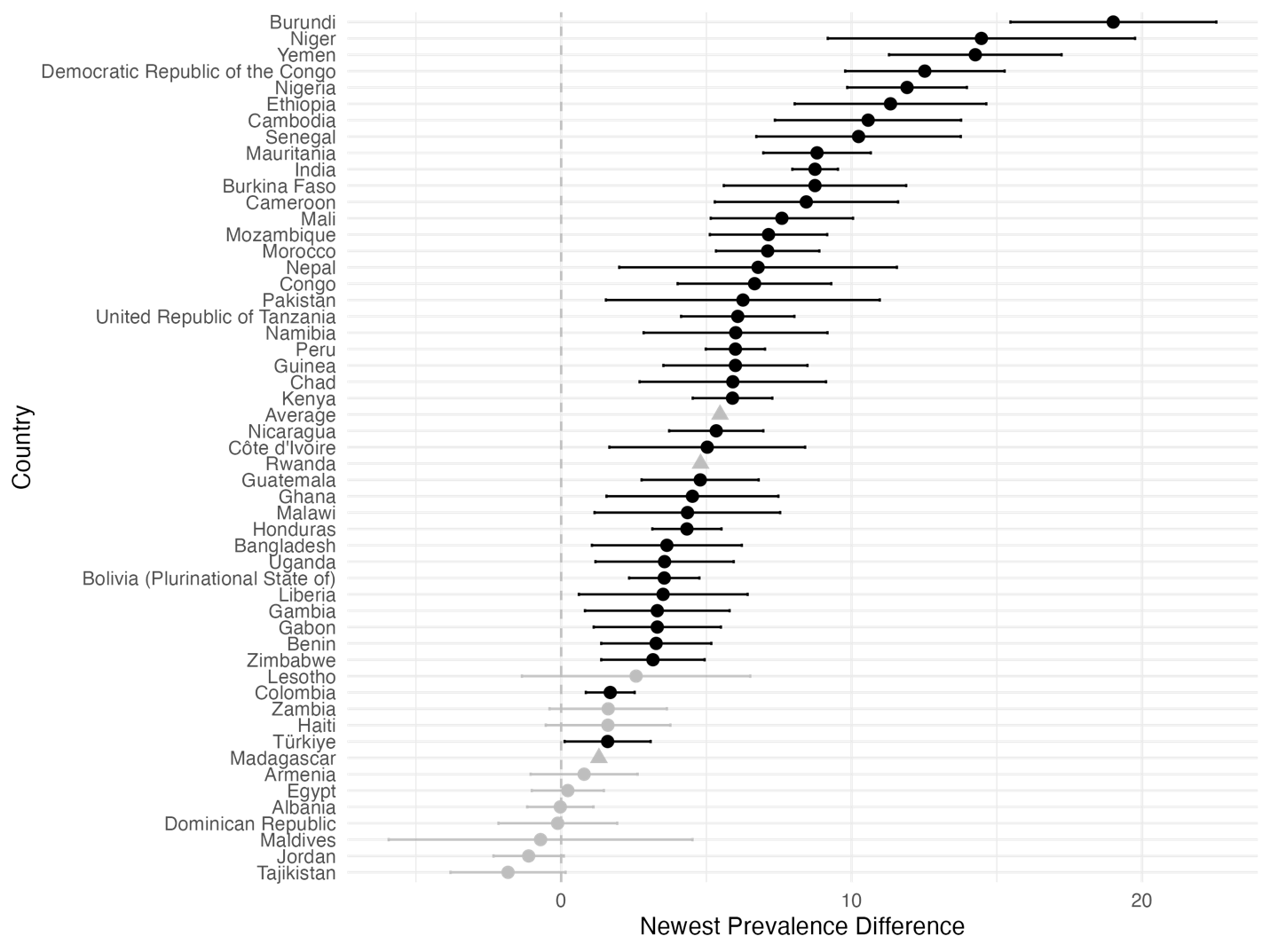


Figure S5. Stunting rural-urban prevalence difference at earliest data point.


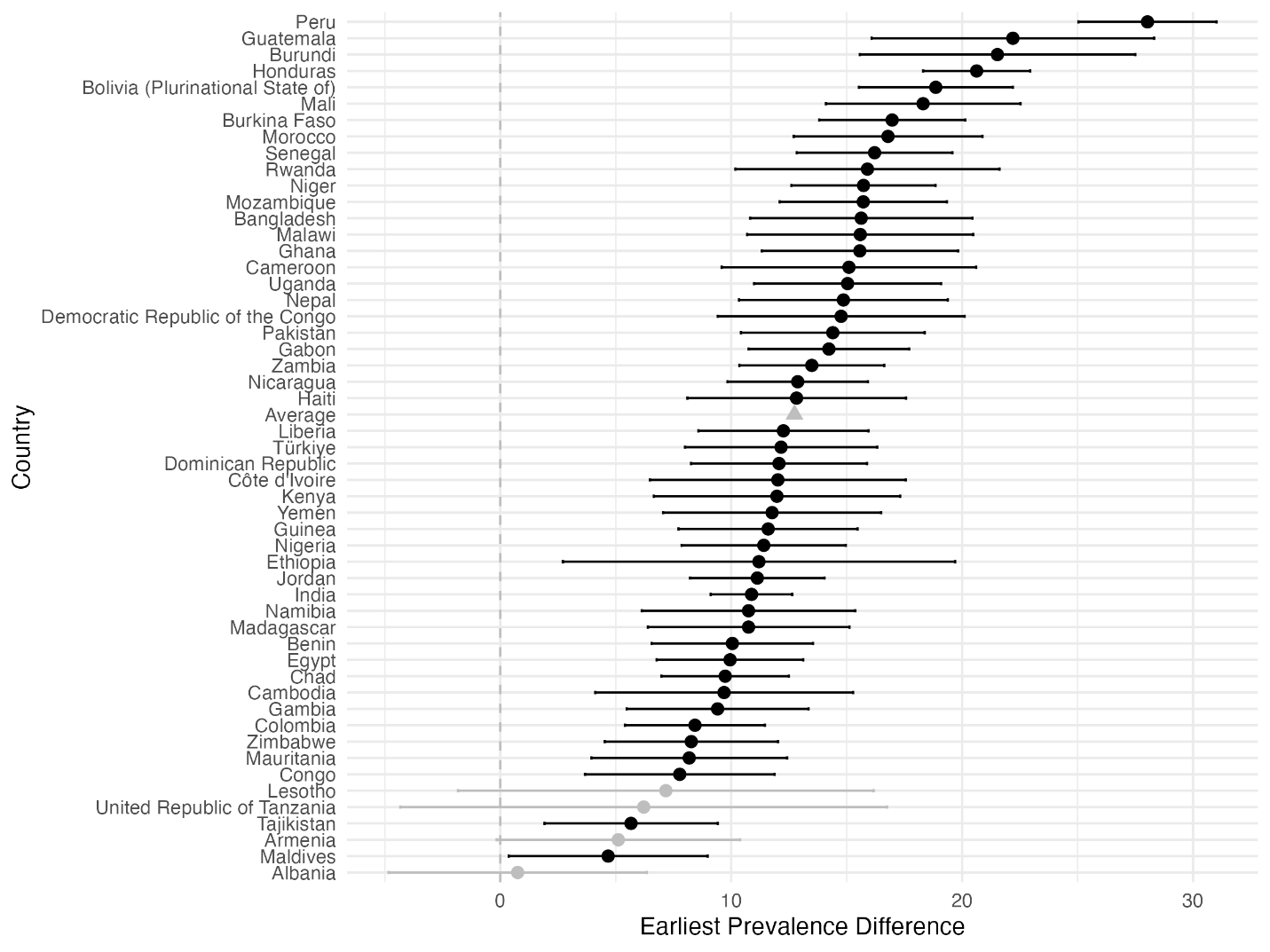


Figure S6. Stunting rural-urban prevalence difference at most recent data point.


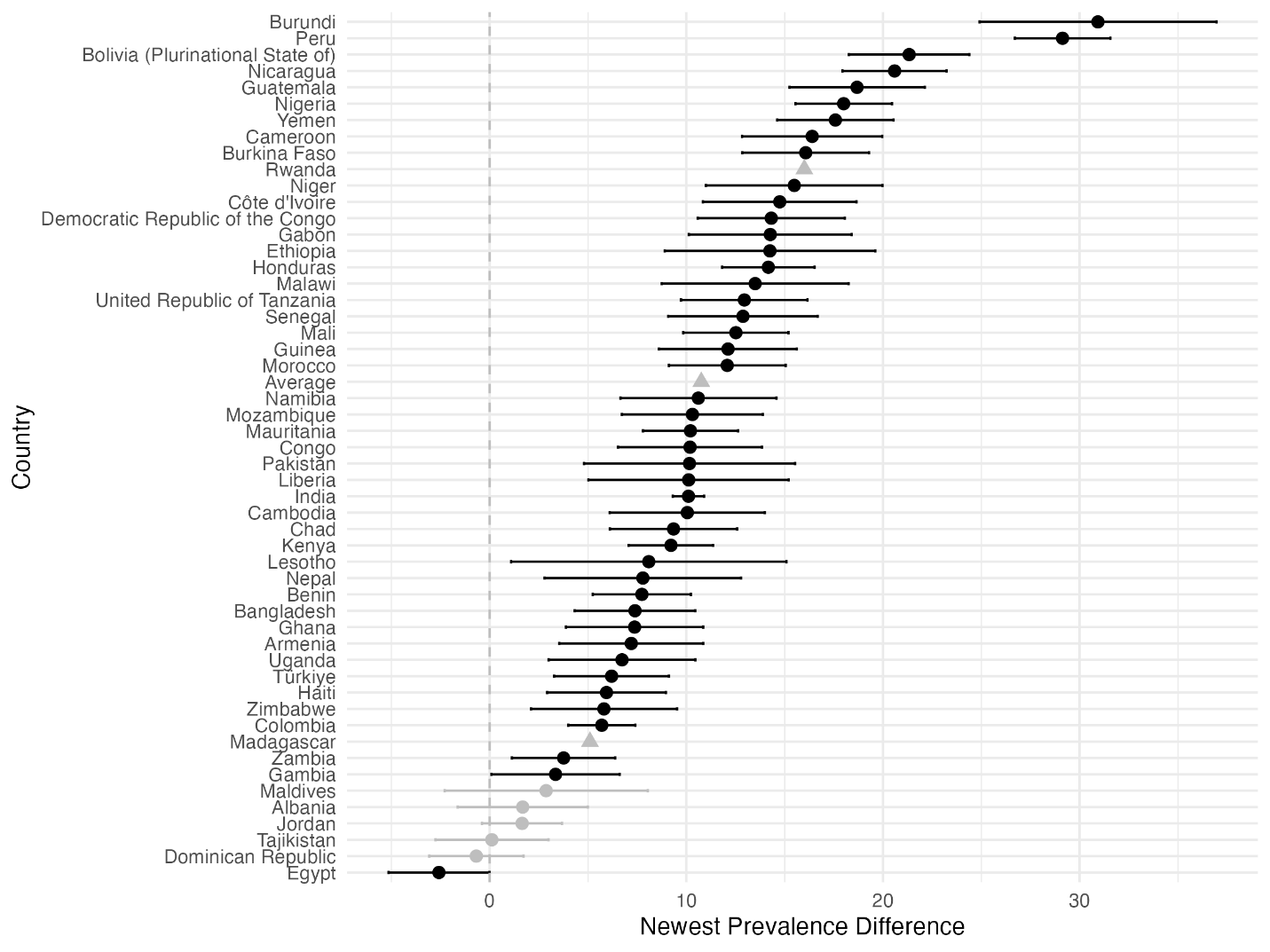


Figure S7. Wasting rural-urban prevalence difference at earliest data point.


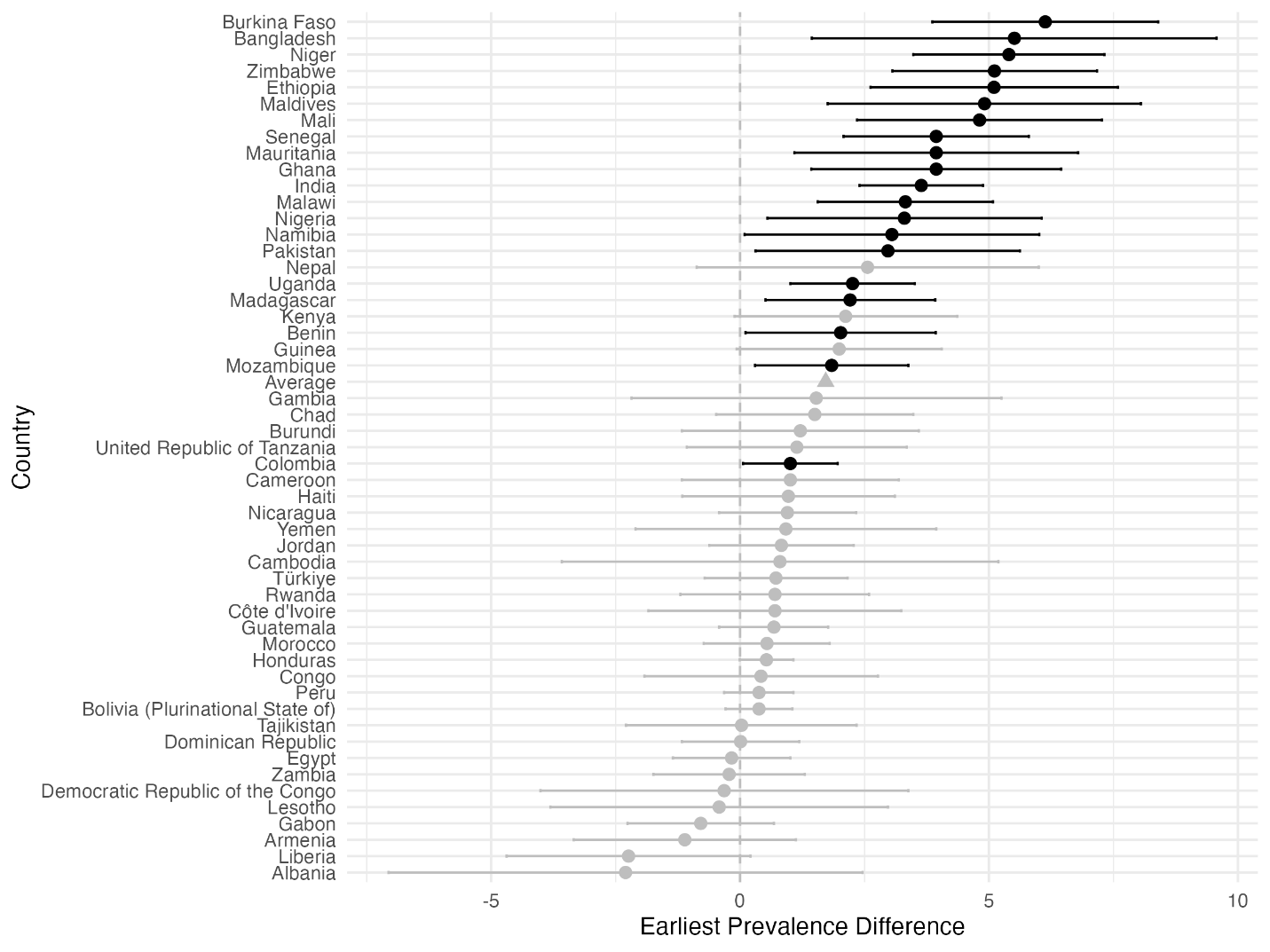


Figure S8. Wasting rural-urban prevalence difference at most recent data point.


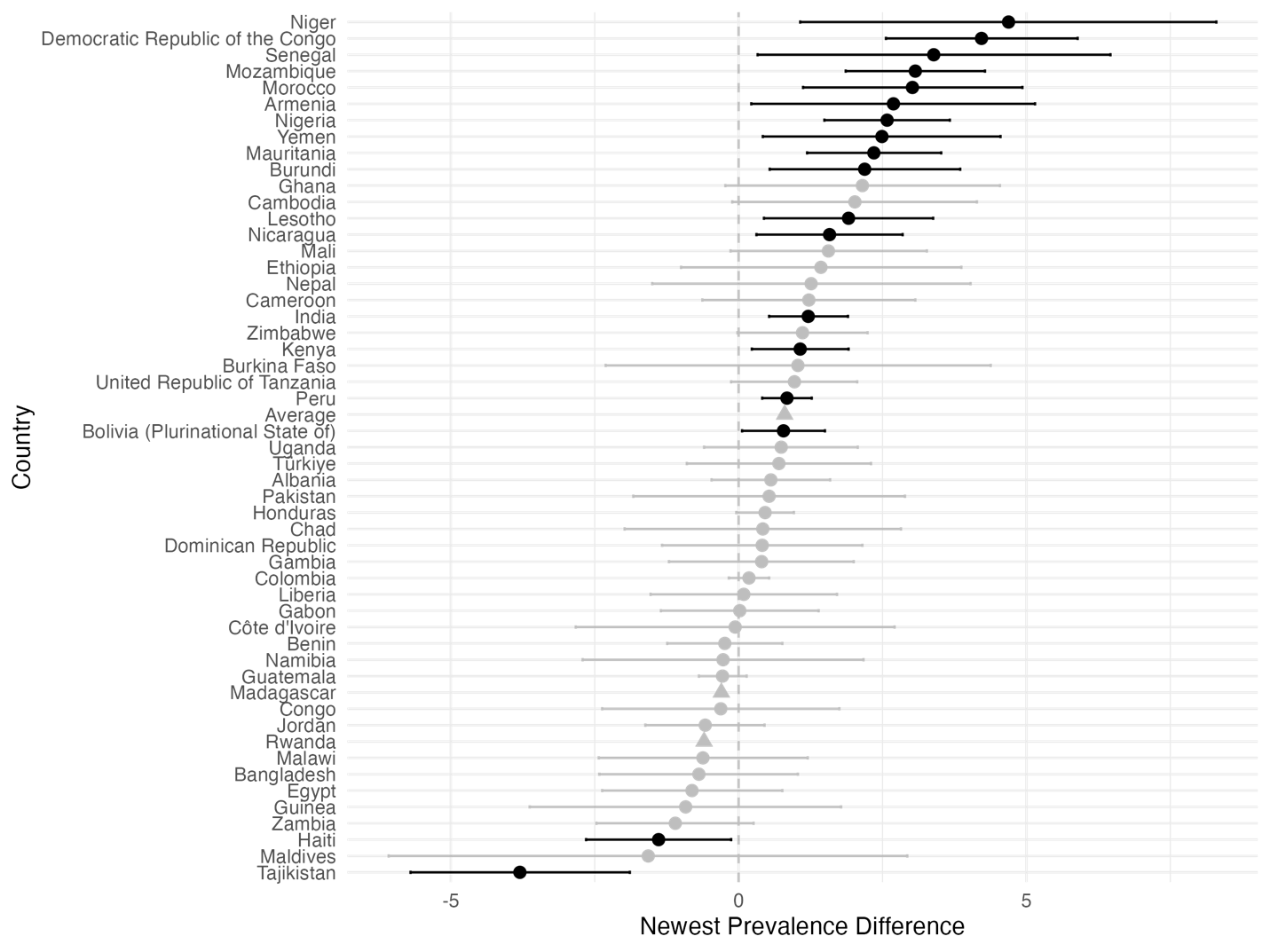


Figure S9. Severe wasting rural-urban prevalence difference at earliest data point.


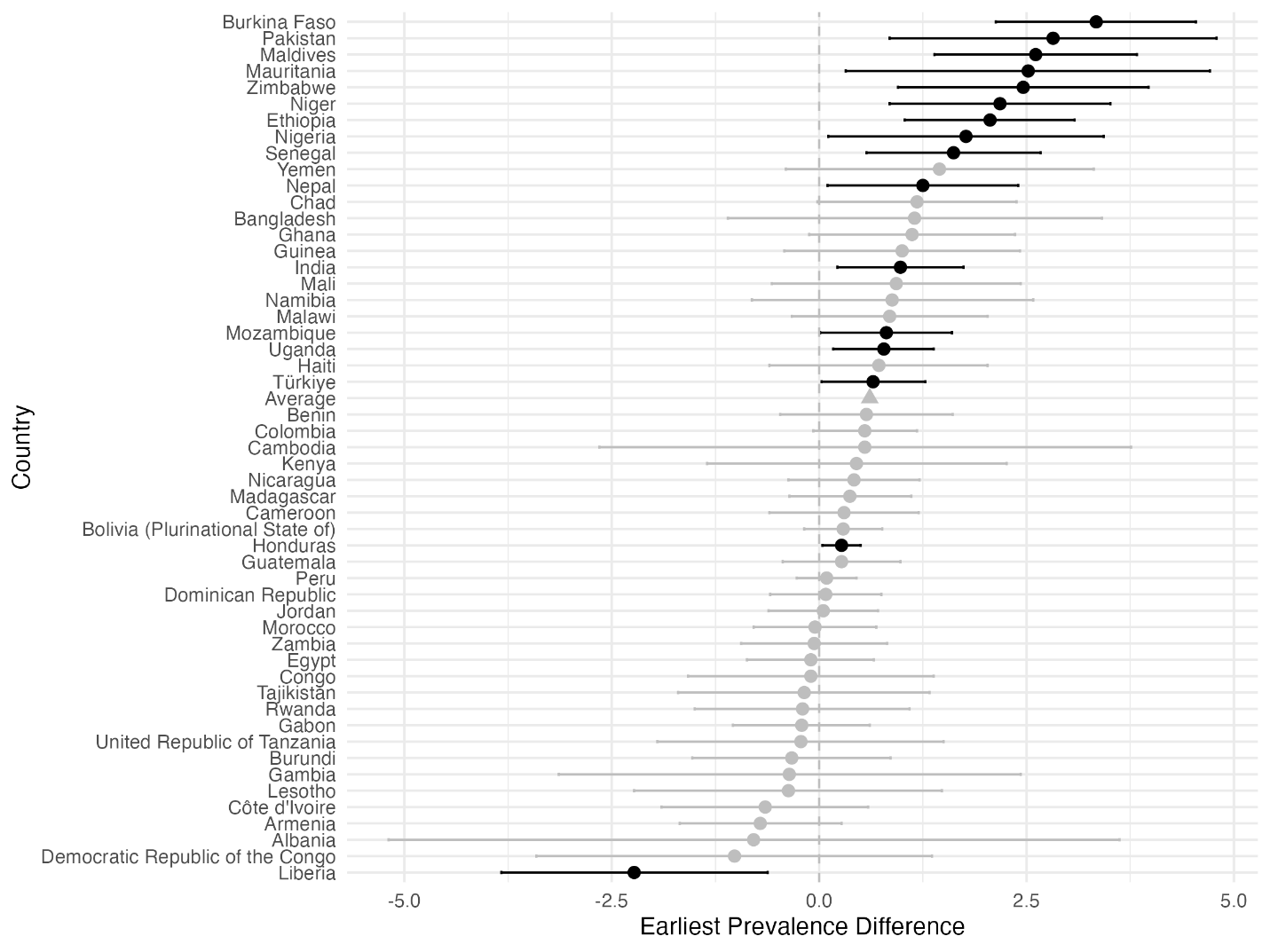


Figure S10. Severe wasting estimated rural-urban difference at most recent data point.


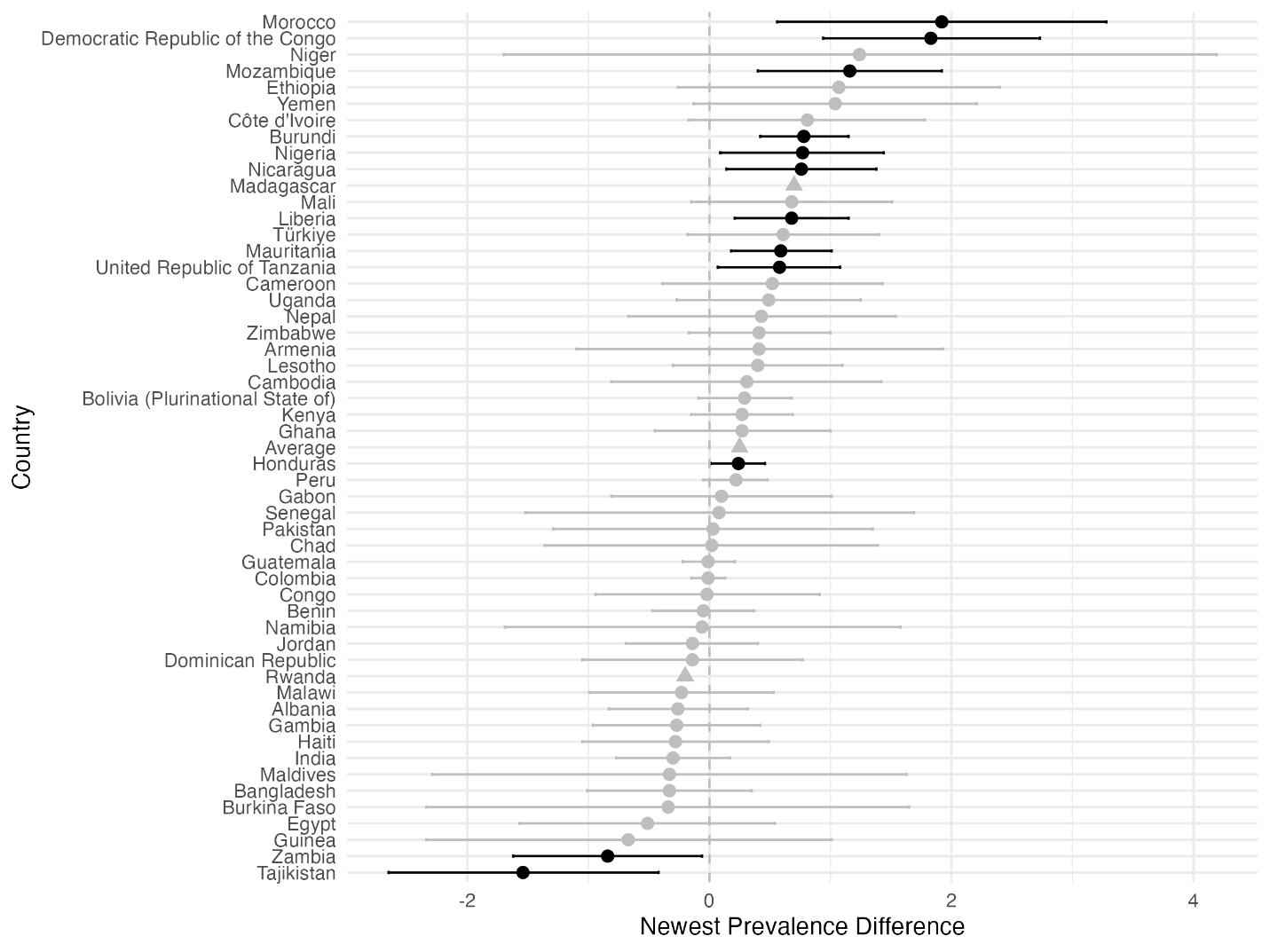

Supplement: Supplementary file 1 — Supplementary Material 1 [file 12939_2026_2798_MOESM1_ESM.docx]
